# Genomic and expression analyses of *Tursiops truncatus* T cell receptor gamma (TRG) and alpha/delta (TRA/TRD) loci reveal a similar basic public γδ repertoire in dolphin and human

**DOI:** 10.1186/s12864-016-2841-9

**Published:** 2016-08-15

**Authors:** Giovanna Linguiti, Rachele Antonacci, Gianluca Tasco, Francesco Grande, Rita Casadio, Serafina Massari, Vito Castelli, Arianna Consiglio, Marie-Paule Lefranc, Salvatrice Ciccarese

**Affiliations:** 1 Department of Biology, University of Bari, via E. Orabona 4, 70125 Bari, Italy; 2 Biocomputing Group, CIRI-Health Science and Technologies/Department of Biology, University of Bologna, via Selmi 3, 40126 Bologna, Italy; 3 Zoomarine Italia SpA, via Casablanca 61, 00071 Pomezia, RM Italy; 4 Department of Biological and Environmental Science e Technologies, University of Salento, via per Monteroni, 73100 Lecce, Italy; 5 CNR, Institute for Biomedical Technologies of Bari, via Amendola, 70125 Bari, Italy; 6 IMGT®, the international ImMunoGeneTics information system®, Laboratoire d’ImmunoGénétique Moléculaire, Institut de Génétique Humaine, UPR CNRS 1142, University of Montpellier, 34396 Montpellier Cedex 5, France

**Keywords:** T cell receptor, TRG locus, TRGV, TRGJ and TRGC genes, TRA/TRD locus, TRAV and TRDV genes, Dolphin genome, Expression analysis, IMGT

## Abstract

**Background:**

The bottlenose dolphin (*Tursiops truncatus*) is a mammal that belongs to the Cetartiodactyla and have lived in marine ecosystems for nearly 60 millions years. Despite its popularity, our knowledge about its adaptive immunity and evolution is very limited. Furthermore, nothing is known about the genomics and evolution of dolphin antigen receptor immunity.

**Results:**

Here we report a evolutionary and expression study of *Tursiops truncatus* T cell receptor gamma (TRG) and alpha/delta (TRA/TRD) genes. We have identified *in silico* the TRG and TRA/TRD genes and analyzed the relevant mature transcripts in blood and in skin from four subjects.

The dolphin TRG locus is the smallest and simplest of all mammalian loci as yet studied. It shows a genomic organization comprising two variable (V1 and V2), three joining (J1, J2 and J3) and a single constant (C), genes. Despite the fragmented nature of the genome assemblies, we deduced the TRA/TRD locus organization, with the recent TRDV1 subgroup genes duplications, as it is expected in artiodactyls.

Expression analysis from blood of a subject allowed us to assign unambiguously eight TRAV genes to those annotated in the genomic sequence and to twelve new genes, belonging to five different subgroups. All transcripts were productive and no relevant biases towards TRAV-J rearrangements are observed.

Blood and skin from four unrelated subjects expression data provide evidence for an unusual ratio of productive/unproductive transcripts which arise from the TRG V-J gene rearrangement and for a “public” gamma delta TR repertoire. The productive cDNA sequences, shared both in the same and in different individuals, include biases of the TRGV1 and TRGJ2 genes.

The high frequency of TRGV1-J2/TRDV1- D1-J4 productive rearrangements in dolphins may represent an interesting oligo-clonal population comparable to that found in human with the TRGV9- JP/TRDV2-D-J T cells and in primates.

**Conclusions:**

Although the features of the TRG and TRA/TRD loci organization reflect those of the so far examined artiodactyls, genomic results highlight in dolphin an unusually simple TRG locus. The cDNA analysis reveal productive TRA/TRD transcripts and unusual ratios of productive/unproductive TRG transcripts. Comparing multiple different individuals, evidence is found for a “public” gamma delta TCR repertoire thus suggesting that in dolphins as in human the gamma delta TCR repertoire is accompanied by selection for public gamma chain.

**Electronic supplementary material:**

The online version of this article (doi:10.1186/s12864-016-2841-9) contains supplementary material, which is available to authorized users.

## Background

Bottlenose dolphin (*Tursiops truncatus*) and the other cetaceans represent the most successful mammalian colonization of the aquatic environment and have undergone a radical transformation from the original mammalian bodyplan. The discovery of two archaic whales with morphological homology between Cetacea and Artiodactyla brought conclusive anatomical support to clade Cetartiodactyla [1, 2]. Whales and hippos shared a common semiaquatic ancestor that branched off from other artiodactyls around 60 million years ago [3–5]. One of the two branches would evolve into cetaceans, possibly beginning about 52 million years ago, with the protowhale *Pakicetus*, which underwent aquatic adaptation into the completely aquatic cetaceans [3]. So far nothing is known about the genomic organization of dolphin immunoglobulins (IG) and T cell receptor (TR) loci. The only studies of antigen receptors immunity revealed that IgG are present in whales [6, 7] and IGHG and IGHA genes have been described in the Atlantic bottlenose dolphin [8]. Within artiodactyls, the locus organization and expression of TRG and TRA/TRD genes have been characterized in ruminants; these species have been shown to possess a large TRG [9–11] and TRA/TRD [12–14] germline repertoire.

Here we present a evolutionary and expression analysis of *Tursiops truncatus* TRG and TRA/TRD genes. The surprising feature concerning TRG genes was, on the one hand, that the overall organization of the dolphin TRG locus resembles more the structure of a typical cassette of artiodactyls (IMGT®, the international ImMunoGeneTics information system®, http://www.imgt.org [15] > Locus representation: Sheep (*Ovis aries*) TRG1) than the structure typical of the human locus (IMGT® > Locus representation: Human (*Homo sapiens*) TRG). On the other hand, equally surprising was the finding of an unusual mechanism of biases in the V-J gene rearrangement usage, which is reminiscent of the most frequently used in the human peripheral γδ T cells repertoire of productively rearranged TRGV genes [16]. Despite the fragmented and incomplete nature of the assembly, we have obtained important information on the genomics and the evolution of the TRA/TRD dolphin potential repertoire and its relationship with the expressed chains. Furthermore, the structural 3D visualization, computed by adopting a comparative procedure, using cDNA TRGV-J and TRDV-D-J rearranged amino acid sequences from a single individual, is consistent with the finding that the predicted γδ pairing, present both in the blood and in the skin, is shared among the organisms living in a controlled environment (kept under human care) as well as in those living in marine environment. This finding highlights in dolphin the existence of a basic “public” γδ repertoire of a given TR in a range of public T cell responses.

## Results

### Genomic arrangement and evolution of the dolphin TRG locus

**Fig. 1:**
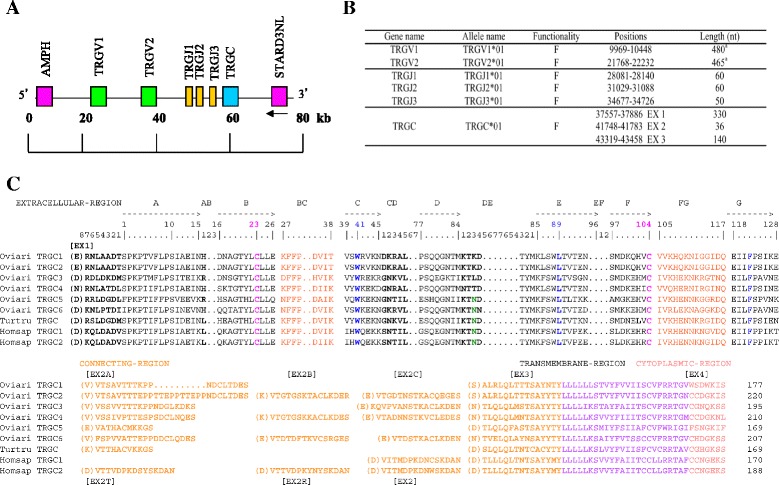
Schematic representation of the genomic organization of the dolphin TRG locus as deduced from the genome assembly Ttru_1.4. **a** The diagram shows the position of all V, J and C genes according to the IMGT® nomenclature. The AMPH (located 15.5 kb upstream of the first TRGV gene) and the STARD3NL (located 11.6 kb downstream of the unique TRGC gene, in the inverted transcriptional orientation) genes at the 5′ end and at the 3′ end, respectively of the TRG locus are shown. Boxes representing genes are not to scale. Exons are not shown. **b** Description of the TRGV, TRGJ, and TRGC genes in the dolphin genome. The position of all genes in the JH473572.1 scaffold and their classification are reported. The bottlenose dolphin (*Tursiops truncatus*) TRG genes and alleles have been approved by the WHO/IUIS/IMGT nomenclature subcommittee for IG, TR and MH [66, 67]. ^a^ From L-PART1 to 3′ end of V-REGION. **c** IMGT Protein display of the dolphin, human and sheep TRGC genes. The description of the strands and loops is according to the IMGT unique numbering for C-DOMAIN [68]. The extracellular region is shown with black letters, the connecting region is in orange, the transmembrane-region is in purple, and the cytoplasmic region is in pink. 1st-CYS C23, CONSERVED-TRP W41 and hydrophobic AA L89 and 2nd-CYS C104 are colored (IMGT color menu) and in bold

The recent availabily of a high quality draft sequence of the bottlenose dolphin (*Tursiops truncatus*) genome [17] (BioProject: PRJNA20367) allowed us to identify the dolphin TRG locus in two overlapping scaffolds (GEDI ID: JH473572.1; BCM-HGSC ID: contig 425448–578749) that provided a genomic sequence assembly of 188.414 kb (gaps included). In the dolphin, as in all mammalian species so far studied [18–20], the amphiphysin (AMPH) gene flanks the TRG locus at its 5′ end and the related to steroido-genic acute regulatory protein D3-N-terminal like (STARD3NL) gene flanks the TRG locus at its 3′ end. We annotated all the identified dolphin TRG genes using the human (GEDI ID: AF159056) and ovine (GEDI ID: DQ992075.1, DQ992074.1) TRG genomic sequences as a reference; the beginning and end of each coding exon were accurately identified by locating the splice sites and the flanking recombination signal (RS) sequences of the V and J genes (Fig. 1). According to our results, the dolphin locus is the simplest of the mammalian TRG loci identified to date (Additional file 1) [9, 10, 21–23]. It spans only 48 kb and its genes are arranged in a pattern comprising 2 TRGV, 3 TRGJ genes and a single TRGC (Additional file 2) gene. A closer inspection of the dolphin, human and sheep constant genes (Fig. 1c), reveals, that the dolphin TRGC (Additional file 2) gene possesses a single small exon 2 (EX2) which is more similar to the sheep TRGC5 EX2 than to the human TRGC1 EX2 (whereas in contrast the human TRGC2 gene has polymorphic duplicated or triplicated exons 2 [24]) (Fig. 1c). The dotplot matrix of dolphin TRG and sheep TRG1 loci genomic comparison displays a remarkable consistency of the identity diagonals, from the sheep TRGV11-1 gene to the TRGC5 gene (red rectangle in Additional file 3A) with a remarkable compactness of the three J genes. Therefore the overall organization of the dolphin TRG locus resembles more the structure of a typical single cassette of artiodactyls (IMGT®, the international ImMunoGeneTics information system®, http://www.imgt.org [15] > Locus representation: Sheep (*Ovis aries*) TRG1), than the structure typical of the human locus (IMGT® > Locus representation: Human (*Homo sapiens*) TRG) (Additional file 3B). High bootstrap values (97 to 100) in the phylogenetic tree from artiodactyls (sheep and dromedary), human and dolphin, grouped dolphin TRGV1 gene with human TRGV9 gene and sheep TRGV11-1 (a pseudogene), and dolphin TRGV2 gene with human TRGV11 (an ORF) and sheep TRGV7 gene (Additional file 4A). It is noteworthy that, in sheep the TRGV11-1 and the TRGV7 genes lie within the TRGC5 cassette (Additional file 4), previously shown to be the most ancient one in cattle and sheep [9].

### Genomic arrangement and evolution of the dolphin TRA/TRD genes

**Fig. 2:**
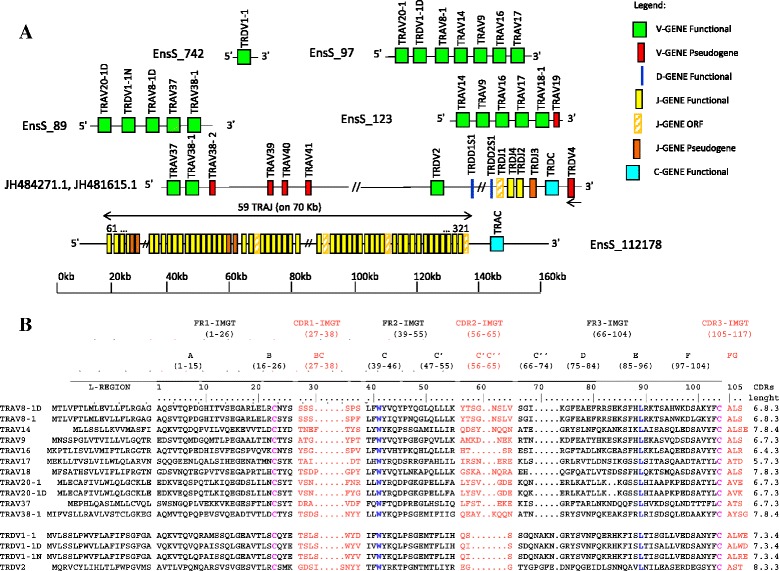
Schematic representation of the genomic organization of the bottlenose dolphin (*Tursiops truncatus*) TRA/TRD locus as deduced from the genome assembly Ttru_1.4. **a** The diagram shows the position of the TRDV, TRDD, TRDJ and TRDC genes and of the TRAV, TRAJ and TRAC genes according to the IMGT® nomenclature. The retrieval of the relevant contigs from the GenBank (JH484271.1 and JH481615.1) and Ensembl databases (S_742; S_97; S_89; S_123 and S_112178), has allowed the identification, starting from the 5′ end of the locus, of 16 TRAV (including 5 pseudogenes), 5 TRDV (including 1 pseudogene), 2 TRDD, 4 TRDJ (including one ORF and 1 pseudogene), 1 TRDC, 59 TRAJ and 1 TRAC genes, located in a genomic region spanning approximatively 450 Kb (Additional file 5). Boxes representing genes are not to scale. Exons are not shown. Arrow head indicates the transcriptional orientation of the TRDV4 gene. The arrows above the line of the TRAJ genes indicate the 70 kb region that has been magnified in the lower part of the figure. **b** IMGT Protein display for the dolphin TRAV and TRDV functional genes. The description of the strands and loops is according to the IMGT unique numbering for V-REGION [25]. 1st-CYS C23, CONSERVED-TRP W41, hydrophobic L89 and 2nd-CYS C104 are colored (IMGT color menu) and in bold

Analysis of the Ttru_1.4 dolphin genome assembly confirmed that the TRD genes are clustered within the TRA locus, as in eutherians and birds. The organization and structure of the dolphin TRA/TRD locus is similar to the organization of the locus in humans (IMGT®, Locus representation human (*Homo sapiens*) TRA/TRD) [16], i.e. the TRAV genes are located at its 5′ end with TRDV interspersed, followed by the TRDD genes (3 in humans and at least 2 in the dolphin), the TRDJ genes (four in both species), and by a single TRDC gene (Additional file 5). A single TRDV gene located, as in all mammals, in an inverted transcriptional orientation downstream of the TRDC gene, has been named TRDV4 by homology with the other artiodactyl TRA/TRD loci although no TRDV3 gene has yet been isolated in dolphin (Fig. 2a). This gene in dolphin, in contrast to all the so far analyzed species, is a pseudogene, since we have found both by *in silico* analysis and by PCR on genomic DNA, the presence of a stop codon (Additional file 6). Figure 2b shows the amino acid sequences of the dolphin TRA/TRD variable genes aligned according to the IMGT unique numbering for V domain [25]. Evolutionary analysis of dolphin, sheep, and human TRAV is shown in Additional file 7A. The tree shows that 10 dolphin subgroups form a monophyletic group with a corresponding human and sheep gene subgroup, consistent with the occurrence of distinct subgroups prior to the divergence of the three mammalian species. Three human TRAV subgroup (TRAV19, TRAV20 and TRAV40) were found in dolphin, and not in sheep. In the TRA/TRD locus, the TRDJ, TRDD, and TRDC genes are followed finally by the TRAJ genes (Additional file 8 and Additional file 9), 61 in humans and 59 in dolphin, and by a single TRAC. Also in this case, the presence of several variable genes (TRDV1-1, TRDV1-1D and TRDV1-1 N) belonging to the TRDV1 subgroup, scattered in three different contigs, makes this portion of the dolphin locus more similar to the TRA/TRD locus of artiodactyls than to the human locus, as humans have only a single TRDV1 gene [16], while in cattle [13, 26], sheep [12] and other artiodactyls the TRDV1 subgroup is a large, multigene subgroup. In the phylogenetic tree, the membership of the TRDV1 genes is supported by the monophyletic groupings, which are marked by 25 sheep, 6 dromedary and 3 dolphin members in contrast with the single human one (Additional file 7B).

### 5′ RACE PCR and RT-PCR on blood and skin RNA identified the dolphin TRG, TRA and TRD chains repertoire

**Fig. 3:**
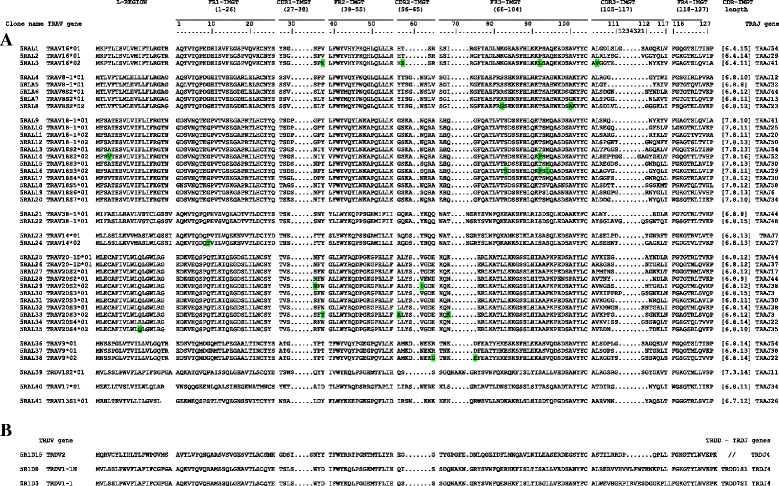
IMGT Protein display of the TRA (**a**) and TRD (**b**) cDNA clones. The TRAV and TRAJ genes are listed respectively at the left and the right of the figure. Leader region (L-Region), complementary determining regions (CDR-IMGT) and framework regions (FR-IMGT) are also indicated, according to the IMGT unique numbering for V-REGION [25]. The TRAV allele amino acid changes, if any are green boxed. The name of the clones are also reported. The TRDV and TRDJ genes are listed respectively at the left and the right of the figure. In (**b**), for TRDV2, TRDV1-1 N and TRDV1-1, the CDR-IMGT lengths are of [8.3.13], [7.3.20] and [7.3.21], respectively. 5R1D15 clone (TRDV2) lacks the TRDD gene. 5R1D3 clone (TRDV1-1) has a new TRDD gene (TRDD7S1) with respect to the available genomic sequence. The name of the clones are also reported

**Fig. 4:**
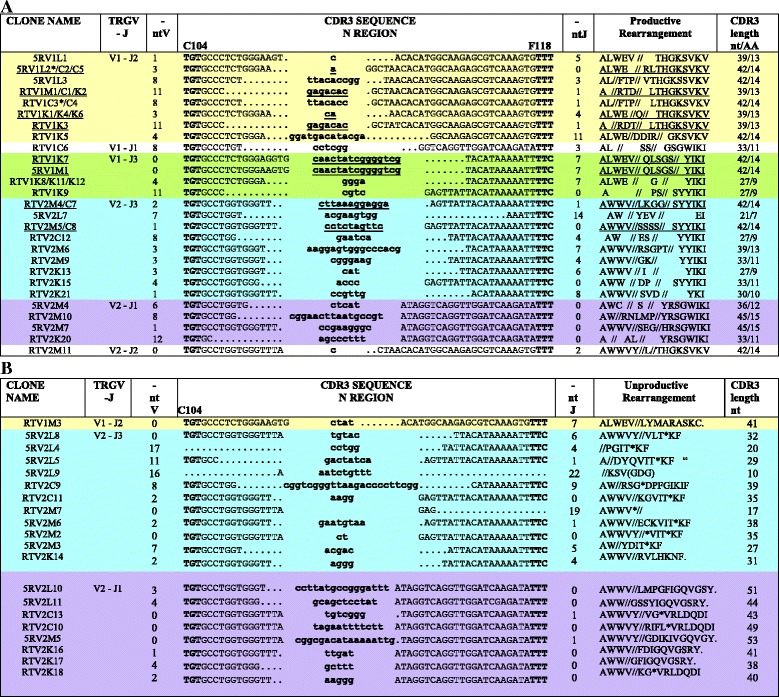
CDR3-IMGT nucleotide sequences retrieved from the cDNA clones with productive (**a**) and unproductive (**b**) rearrangements. Nucleotide sequences are shown from codon 104 (2nd-CYS) to codon 118 (J-PHE) (**a** and **b**). N-nucleotides added by the deoxynucleotidyltransferase terminal (DNTT, TdT) are indicated in lower cases. Numbers in the left and right columns indicate the number of nucleotides that are trimmed from the 3′V-REGION and 5′J-REGION, respectively; the germline region of the TRGV and TRGJ genes coincides with 0 in the nt V and nt J columns, respectively. In A, clonotypes with the same CDR3-IMGT nucleotide sequence deriving from two or more animals (letters M, L, K and C) are underlined. A shared clonotype (AA) between individuals has per definition a given V and J gene and allele and a given AA sequence for the junction. Three individuals (M, K and C) share the same CDR3 (AA) sequence with the V1-J2 rearrangement (RTV1M1/C1/K2/K3) however the junction in K3 differs from the junction in the other shared clonotypes by a nucleotide difference in the 5′J-REGION which may represent an allele of the TRGJ2 gene. Similarly, for the K and M shared clonotypes with a V1-J3 rearrangement (RTV1K7/5RV1M1), the junction in M1 differs from the junction in K7 by a nucleotide in the 3′V-REGION, which may represent an allele of the TRGV1 gene. This has been described as “convergent recombination” in which a given “public” TR amino acid sequence may be encoded by different nucleotide sequences both within the same and in different individuals [28]. In B, unproductive rearrangements (non-redundant out-of-frame clonotypes column of Table 2) for the presence of a stop codon (*) and for frameshifts in the CDR3, are indicated

Four types of 5′ RACE and three types of RT-PCR (total of 6 and 6 experiments, respectively) on total RNA isolated from the peripheral blood of three unrelated adult animals (identified as M, K and L) and from the skin of animal identified by letter C (Tables 1 and 2) were carried out to investigate the dolphin TRG, TRA and TRD chains repertoire. We obtained a total of 105 unique (5RV and RTV) clonotypes of different length, each containing rearranged VJ-C (for TRG and TRA) and V-D-J-C (for TRDV) transcripts. A clonotype (AA) (AA for amino acid) is identified by a given rearranged V gene and allele, a given J gene and allele and a unique amino acid junction [27]. The V domains were checked for their typical features, i.e., the leader region and the five conserved amino acids (1st-CYS C23, CONSERVED-TRP W41, hydrophobic 89 (here, leucine L89), 2nd-CYS 104 and anchor 118 (J-PHE 118 or J-TRP) characteristic of a V-DOMAIN [25]. The functionality of each clonotype was determined based on the IMGT® criteria: transcripts were considered as productive if they had in-frame junctions and no stop codons, whereas transcripts were considered as unproductive if they had frameshifts and/or stop codons. The junctions comprise the CDR3-IMGT and the two anchors C104 (2nd-CYS) and F118 (J-PHE for TRG, TRA and TRD) or W118 (for one TRAJ, identified in this study as TRAJ34) (Fig. 3 and Additional file 9). Fifty-nine TRG clonotypes were obtained from M, L, K and C, respectively. Twenty of the 59 TRG clonotypes contained out-of-frame cDNAs (Table 2). All the remaining 39 TRG clonotypes were productive, containing in-frame cDNA sequences and were submitted to and accepted by the ENA database in which they are identified by the HG328286 to HG328324 Accession numbers (Table 2 and Additional file 10). As all possible rearrangements between the two TRGV and the three TRGJ genes were found both in blood and in skin, it can be concluded that all dolphin TRG genes contribute to the formation of productive transcripts in all six TRGV-TRGJ combinations (Fig. 4).

**Table 1:** List of primers used in 5′ RACE, RT and genomic PCR

Locus	Primer	Genomic Orient.^a^	Sequence 5′–3′	Primer length	Location and sequence positions	Description
TRG	TC3L	REV	TGAGGAGGAGAAGGAGGT	18-mer	TRGC EX3^b^ 43364–43381	5′RACE, RT-PCR
	TC1L1	REV	GACGATACATACGAGTTCA	19-mer	TRGC EX1^b^ 37807–37825	dC-TAILED cDNA
	TC1L3	REV	AAGGCAAAGATGTGTTCCAG	20-mer	TRGC EX1^b^ 37635–37654	nested, RT-PCR
	TC1L2	REV	TGTTGCCATTCTTTTCTTTCC	21-mer	TRGC EX1^b^ 37692–37712	nested, V1-V2 RT-PCR
	TV1LU	FWD	GCTCGCTCTGACAGTCCTT	19-mer	TRGV1 L-Part1^b^ 9989–10007	V1 RT-PCR, V1J2 genomic PCR
	TV7LU	FWD	GATCCTCTTCTCCTCCCTCTG	21-mer	TRGV2 L-Part1^b^ 21785–21805	V2 RT-PCR, V2J3 genomic PCR
	J2GL	REV	TGACGCTCTTGCCATGTGTT	20-mer	TRGJ2^b^ 31033–31052	V1J2 genomic PCR
	J5BR	REV	CGGCGATGGGACAAAACTTG	20-mer	TRGJ3^b^ 34698–34717	V2J3 genomic PCR
TRA	TA1C1L	REV	GAAGGTCTGGTTGAAGGTG	19-mer	TRAC EX1^c^ 86762–86780	5′RACE, RT-PCR
	TA1C2L	REV	TGTCTCCGCATCCCAAATC	19-mer	TRAC EX1^c^ 86745–86763	dC-TAILED cDNA
	TA1C3L	REV	TGCTGGATTTGGGGCTTCT	19-mer	TRAC EX1^c^ 86574–86592	nested PCR
TRD	TD1C1L	REV	AGAACTCCTTCACCAGAC	18-mer	TRDC EX1^d^ 85917–85934	5′RACE, RT-PCR
	TD1C2L	REV	CTTATAGTTACATCTTTGGG	20-mer	TRDC EX1^d^ 85938–85957	dC-tailed cDNA
	TD2CL	REV	CTGGAGTTTGAGTTTGATT	19-mer	TRDC EX2^d^ 86773–86791	nested PCR
	VD4U	FDW	GTGGAAGGTTTTGTGGGTCAGG	22-mer	TRDV4 EX^d^ 91850–91871	V4 genomic PCR
	VD4L	REV	TAACCAAGTGACCCAGATTT	20-mer	TRDV4 EX^d^ 92062–92081	V4 genomic PCR

^a^FWD: forward orientation, REV: reverse orientation (IMGT, Genomic orientation, http://www.imgt.org/IMGTindex/Orientation.php)

^b^Acc. Number: JH473572.1

^c^Acc. Number: EnsS_112178

^d^Acc. Number: JH481615.1

**Table 2:** Summary of the different 5′RACE and RT-PCR experiments and the obtained rearrangement types

Locus	Animal tissue	Experiment	FWD primer name	REV primer name	Total number of non-redundant clonotypes	Number of non-redundant out-of-frame clonotypes	Number of non-redundant in-frame clonotypes	Non-redundant in-frame clonotypes by rearrangement type	GenBank (GEDI) accession numbers
TRG	Blood M	5′RACE	--	TC3L/TC1L1/TC1L2	7^a^	(7) 4	3	1 TRGV1*01–TRGJ3*01	HG328298
								2 TRGV2*01–TRGJ1*01	HG328299/300
	Blood M	RT-PCR	TV1LU	TC3L/TC1L2	8	(8) 1	7	1 TRGV1*01–TRGJ2*01	HG328291
			TV7LU	TC3L/TC1L2				4 TRGV2*01–TRGJ3*01	HG328292/93/94/95
								1 TRGV2*01–TRGJ1*01	HG328296
								1 TRGV2*01–TRGJ2*01	HG328297
	Blood L	5′RACE	--	TC3L/TC1L1/TC1L2	11^a^	(11) 6	5	2 TRGV1*01–TRGJ2*01	HG328286/8
								1 TRGV1*02–TRGJ2*01	HG328287
								2 TRGV2*01–TRGJ3*01	HG328289/90
	Blood K	RT-PCR	TV1LU	TC3L/TC1L2	20	(20) 5	15	6 TRGV1*01–TRGJ2*01	HG328305/7/8/10/11/14
			TV7LU	TC3L/TC1L2				5 TRGV1*01–TRGJ3*01	HG328306/9/12/13/15
								3 TRGV2*01–TRGJ3*01	HG328301/02/04
								1 TRGV2*01–TRGJ1*01	HG328303
	Skin C	RT-PCR	TV1LU	TC3L/TC1L3	13	(13) 4	9	4 TRGV1*01–TRGJ2*01	HG328316/17/18/19
			TV7LU	TC3L/TC1L3				1 TRGV1*02–TRGJ2*01	HG328320
								1 TRGV1*02–TRGJ1*01	HG328321
								3 TRGV2*01–TRGJ3*0	HG328322/3/4
TRD	Blood M	5′RACE		TD1C1L/TD1C2L/TD2CL	5^b^	(5) 2	3	1 TRDV1*01–TRDJ4*01	LN610749
								1 TRDV1*01–TRDJ4*01 ^c^	LN610748
								1 TRDV2*01–TRDJ4*01	LN610747
TRA	Blood L	5′RACE		TA1C1L	41	(41) 0	41	^d^	LN610706–LN610746
				TA1C2L					
				TA1C3L					

^a^One clonotype is an incomplete sequence; ^b^One clonotype is a sterile germline transcript; ^c^M TRDV1-1 N -TRDJ4 and TRDV1-1 -TRDJ4 rearrangements have one TRDD gene; ^d^L TRA cDNA clonotypes by rearrangement type are displayed in Fig. 3

To investigate the dolphin TRA chain repertoire, total RNA from the peripheral blood of a female dolphin (identified as L) was used as template in the single 5′ RACE experiment (Tables 1 and 2). A total of 41 different TRA clonotypes were obtained and sequenced (Fig. 3). All sequences were productive (in-frame junction and no stop codon), and the leader region was of 17 to 20 amino acids depending on the V subgroup. The CDR1- and CDR2-IMGT lengths of the transcripts [6.4.], [6.8.], [7.8.] corresponded to nine different TRAV subgroups and 29 different genes and were associated with diverse CDR3-IMGT of various length from 8 to 16 AA. In our cDNA collection, 8 TRAV genes (TRAV16, TRAV8-1, TRAV18-1, TRAV38-1, TRAV14, TRAV20-1D, TRAV9, TRAV17) were assigned unambiguously to genes annotated in the genomic sequence (Fig. 2), while 12 could be assigned to new genes, belonging to five different subgroups. One gene belongs to a new subgroup, TRAV13 (TRAV13S1), not yet identified in the dolphin genomic sequence. One gene belongs to subgroup TRDV1 (TRDV1S2), as shown by its CDR1- and CDR2-IMGT lengths [7.3.], and demonstrates that dolphin TRDV genes can, as in other species, participate to the synthesis of TRA chains by rearranging to a TRAJ gene (here, TRAJ11) [16]. Among the other new genes, six belong to subgroup TRAV45 (TRAV18S2, TRAV18S3, TRAV18S4, TRAV18S5, TRAV18S6, TRAV18S7), three belong to subgroup TRAV20 (TRAV20S2, TRAV20S3, TRAV20S4) and one to subgroup 42 (TRAV8S2). As these last subgroups have several members, an IMGT approved provisional nomenclature was assigned (with the letter S), allowing these genes to be entered in IMGT/GENE-DB and IMGT® tools (IMGT/V-QUEST and IMGT/HighV-QUEST) [15] while waiting for the identification and location of these genes in the reference genomic sequence. Three 5′ RACE experiments on total RNA isolated from the peripheral blood of two unrelated adult animals (identified as M and L) (Tables 1 and 2) were carried out to investigate the dolphin TRD chain repertoire; only one of these three PCR amplifications produced 3 in-frame, 1 out-of-frame and 1 sterile germline, clonotypes from the animal identified as M (Fig. 3b).

### Potential TRGV domain repertoire of productive and unproductive trancripts

Analyzing the TRG in-frame transcripts it is noteworthy that 5 TRG clonotypes were found identical in two or even three different individuals L and C (5RV1L2*/C2/C5), M, C and K (RTV1M1/C1/K2/K3), K and M (RTV1K7/5RV1M1), and M and C (RTV2M4/C7 and RTV2M5/C8) (Additional file 10). This observation was rather intriguing as they represented together 14/39 in-frame sequences whereas in contrast each out-of-frame clonotype was found in a single individual. These shared clonotypes result from V1-J2 rearrangements in L and C (CDR-IMGT lengths [8.7.13]) and in M, C and K (CDR-IMGT lengths [8.7.14]), from V1-J3 rearrangements in K and M (CDR-IMGT lengths [8.7.14]) and from V2-J3 rearrangements in M and C (CDR-IMGT lengths [8.6.14] (Fig. 4 and Additional file 10). This description of shared T cell clonoypes correspond to what is known in the literature as “public T cell response” in which T cells bearing identical TR may respond to the same antigenic epitope in different individuals [28]. Although the number of the germline TRG genes is low, which implies a reduced potential in the V-J recombination, a sufficient diversity and variability of the TR gamma transcripts seems to be guaranteed in the dolphin by the classical process of CDR3 diversity formation during somatic rearrangement [16]. Indeed, the creation of the CDR3 diversity results from the trimming of the 3′V-REGION (up to 12 nucleotides (nt) for the in-frame junctions, up to 17 for the out-of-frame junctions), from the trimming of the 5′J-REGION (up to 14 nt for the in-frame junctions, up to 22 for the out-of-frame junctions), and from the addition at random of the N nucleotides creating the N-REGION (up to 16 nt for the in-frame junctions, up to 23 for the out-of-frame junctions) (Fig. 4). This junction diversity is due to the activity of the terminal deoxynucleotidyl transferase (TdT) encoded by DNTT. The gene (NCBI ID: 101323636) has been identified in the bottle nosed dolphin genome and its amino acid sequence is 84 % identical to the human DNTT. The graphical representation of the number of in-frame versus out-of-frame sequences obtained for the 6 possible TRG rearrangements V1-J1, V1-J2, V1-J3, V2-J1, V2-J2 and V2-J3 display striking differences (Additional file 11). Both tests (Chi-squared *p*-value confirmed with Fisher’s *p*-value) reject the null hypothesis for V1-J2 and V2-J1 (Additional file 12). This result confirms what was noticed at first sight and it follows that V1-J2 gene rearrangements were dominant among the in-frame transcripts and were rare among the out-of-frame transcripts.

**Fig. 5:**
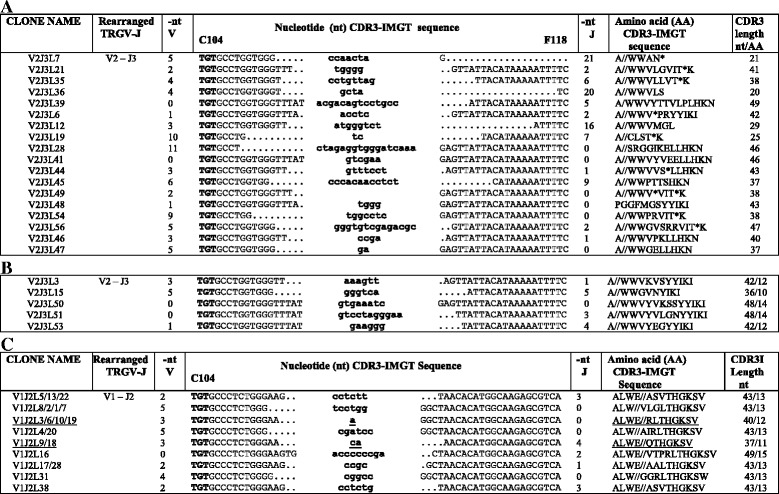
CDR3 nucleotide sequences retrieved from genomic rearranged clones. Nucleotide sequences are shown from codon 104 (2nd-CYS) to codon 118 (J-PHE) (**a** and **b**) and from codon 104 (2nd-CYS) to codon 115 (**c**). They are grouped on the basis of their rearrangement (**a**) and (**b**) TRGV2-TRGJ3 or (**c**) TRGV1-TRGJ2. N-nucleotides added by TdT are indicated in lower cases. Numbers in the left and right columns indicate the number of nucleotides that are trimmed from the 3'V-REGION and 5'J-REGION, respectively. The germline region of the TRGV and TRGJ genes coincides with 0 in the nt V and nt J columns, respectively. Clones with the same CDR3-IMGT nucleotide sequence deriving from two or more animals (L, K and C) are underlined (see also Fig. 4). In (**a**), unproductive rearrangements for the presence of a stop codon (*) and for frameshifts in the CDR3, are indicated

To investigate the high frequency of the out-of-frame rearranged V2-J3 cDNA, genomic PCR was carried out on DNA from blood of the animal identified by L. This choice was motivated both by the ratio of 1 in-frame (5RV2L7) on 4 out-of-frame (5RV2L8, L4, L5 and L9) clonotypes with the rearranged V2-J3 and by the highest number clones (5RV1L1, L2 and L3) with the rearranged V1-J2 (Fig. 4a). The frequency of the out-of-frame V2-J3 genomic rearrangements (Fig. 5a) is in agreement with that of all the respective rearranged cDNA clonotypes (Fig. 4); stop codons in the CDR3 seem to be generated in the unproductive V2-J3 rearrangements during the somatic recombination. Furthermore, genomic V1-J2 clonotypes, obtained by PCR performed on the same animal, demonstrate that they are all productive with two cases of sharing of the CDR3, i.e. V1J2L3/6/10/19 and V1J2L9/18 with 5RV1L2/C2/C5 and RTV1K1/K4/K6 cDNA clonotypes, respectively (Fig. 5c and Additional file 11).

### Computational analyses predict the pairing of the TRGV1-J2 and of the TRDV1-D1-J4 variable domains

**Fig. 6:**
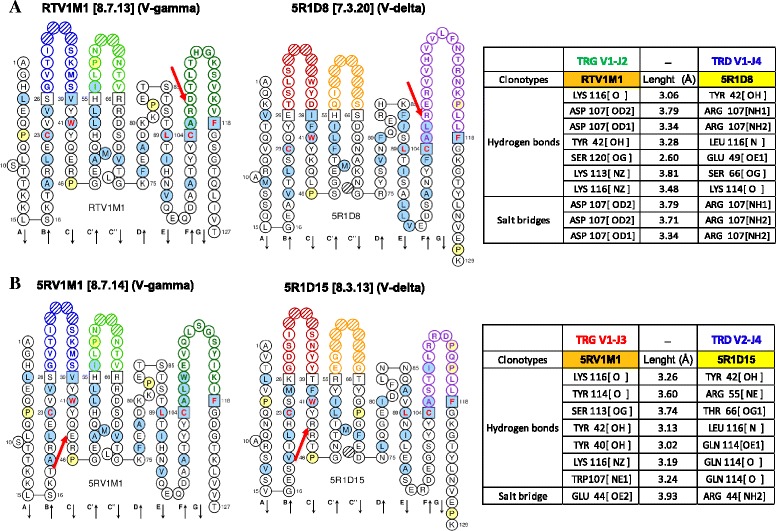
Computationally inferred interaction between RTV1M1 V-gamma domain (TRGV1 – J2) and 5R1D8 V-delta domain (TRDV1-1 N – J4) (**a**) and between 5RV1M1 V-gamma domain (TRGV1 – J3) and 5R1D15 V-delta domain (TRDV2 – J4) (**b**) cDNA clonotypes. In RTV1M1 and 5RV1M1 V-gamma domain CDR-IMGT are blue-green-green; in 5R1D8 and 5R1D15 V-delta domain CDR-IMGT are red-orange-purple. IMGT Collier de Perles of RTV1M1/5R1D8 and 5RV1M1/5R1D15 clones are shown [25, 65]. The protein complex interface were computed by the online tool PDBePISA at the EBI server. (http://www.ebi.ac.uk/msd-srv/prot_int/) and visualized by UCSF Chimera tool (http://www.cgl.ucsf.edu/chimera/) (Additional file 14)

To calculate the most likely computationally inferred interactions between the putative γδ pairing, we analysed the amino acid sequences of the three types of rearranged TRD cDNAs (originated by TRD V1-1 N–D1S1–J4 (5R1D8), V1-1–D7S1–J4 (5R1D3) and V2–J4 (5R1D15) rearrangements, respectively) found in the peripheral blood of the single animal identified in our study with the letter M (Table 2, Fig. 3b), and of three relevant TRG cDNAs (originated by TRG V1–J2, V1–J3 and V2–J3 rearrangements) among the six found in the peripheral blood of the same animal (clones identified by the letter M in Fig. 4a). The comparative inferred interactions of the TRGV1/TRDV1 and TRGV1/TRDV2 V domains, obtained using the AA sequences of the cDNA RTV1M1 (TRGV1–J2) and 5R1D8 (TRDV1-1 N–J4) clonotypes and the sequences of the 5RV1M1 (TRGV1–J3) and 5R1D15 (TRDV2–J4) clonotypes (Fig. 6, Additional file 13), respectively, were computed using as templates the human γδ T cell receptor chains (PDB and IMGT/3Dstructure-DB code: 1hxm) [29, 30]. We point out that, according to visualization of the RTV1M1/5R1D8 complex shown in Additional file 14A, the aspartic acid in the CDR3 position 107 (see IMGT Collier de Perles of the RTV1M1 clone) of the V − gamma domain, deriving from the addition of gagacac nucleotides during the TRG V1-J2 recombination process, is predicted to be significantly involved in the formation of three possible salt bridge(s) and of two of the seven calculated hydrogen bonds with the arginine in position 107 of the 5R1D8 clonotype (V-delta domain) (red arrows in Fig. 6a). The arginine in position 107 derives from the TRDV1-1 N germline sequence in the CDR3 of the TRD V1-J4 rearrangement (‘Protein interfaces, surfaces and assemblies’ service PISA at the European Bioinformatics Institute (http://www.ebi.ac.uk/pdbe/prot_int/pistart.html) [31]. The computationally inferred interaction between the glutamic acid in position 44 in the FR2 of the TRG V1-J3 rearranged cDNA 5RV1M1 clonotype (V-gamma domain) and the arginine in position 44 in the FR2 of the TRD V2-J4 rearranged cDNA 5R1D15 clonotype (V-delta domain) is noteworthy because they are both involved in a possible salt bridge (red arrows in Fig. 6b). The Gln Q114 of the 5R1D15 clonotype is just as important because it is involved in three possible hydrogen bonds with Tyr Y40, Trp W107 and Lys K116 of the 5RV1M1 clone, respectively. In conclusion, we suggest that the RTV1M1/5R1D8 pairing is the most likely to form and it is the most stable (because of its ability to maintain the V-gamma/V-delta domain interactions better) (Fig. 6 and Additional file 13). This consideration, seems to be in compliance with the fact that the TRG V1-J2 rearrangement, found both in the peripheral blood and in the skin, is not only the most frequent among the six possible rearrangements, but it is shared among the organisms living in the same controlled environment (see animals identified by letters K, L and M) as well as in those living in marine environment (see animal identified by letter C) (Fig. 4).

## Discussion

In this study we report an extensive analysis of the genomic organization and expression of the TRG and TRA/TRD genes in dolphin. According to comparative analyses, dolphin TRG locus is the simplest and the smallest among the mammalian TRG loci identified to date [19, 20] and its organization is reminiscent of the structure of a typical single cassette of artiodactyls [9, 15] with a small number of genes, i.e. two TRGV, three TRGJ and one TRGC (Fig. 1).

The analysis of dolphin TRA/TRD locus confirmed that TRD genes are clustered within the TRA locus and that genes belonging to the TRDV1 subgroup are distributed among the TRAV genes as it is commonly expected in artiodactyls TRA/TRD locus [13, 14, 26, 32]. A total of 16 TRAV and 5 TRDV genes have been identified (Fig. 2). By the criterion that gene sequences having 75 % or greater nucleotide identity belong to the same subgroup, the TRAV and the TRDV genes belong to 13 and to three subgroups, respectively (Additional file 7). The sheep TRDV1 subgroup has been estimated to contain at least 40 genes [12], while only 25 TRDV1 genes have been identified in the genomic assembly [14]. The phylogenetic analysis assigns the membership of the dolphin TRDV1 genes due to the monophyletic groupings marked by 25 sheep, 6 dromedary and 3 dolphin members in contrast with the single human one (Additional file 7B).

Dolphin TR alpha chain expression analysis allowed us to identify new TRAV genes, with respect to the available genomic sequence. Furthermore a bias towards rearrangements containing TRA genes belonging to the TRAV18 (12/40 cDNA) and TRAV20 (11/40 cDNA) gene subgroups, was observed (Fig. 3a). On the contrary, the usage of the 61 TRAJ genes is generally random with a slight increase in usage of TRAJ (Fig. 2) between 54 and 22 (31 of 50 functional rearrangements) (Additional file 9); this finding being consistent with the widely accepted view that TRAV-TRAJ recombination proceeds in a coordinated, sequential manner from proximal to progressively more distal TRAV and TRAJ genes [33, 34].

Dolphin TR gamma chain expression analysis demonstrated that the two TRGV and three TRGJ were used in every possible combination, although a bias towards some transcripts (TRGV1-TRGJ2 and TRGV2-TRGJ3) was noted. Furthermore, about half the transcripts using TRGV2 were unproductive due to the presence of stop codons in CDR3. The percentage values of the productive/unproductive rearrangements are similar for both cDNA (Fig. 4) and genomic clones (Fig. 5), in contrast with what is usually obseved (percentage of unproductive rearrangements lower in cDNA, due to nonsense-mediated decay of RNA).

In a previous work [35], it was reported that biased V-J gene rearrangement contributes to the regulation of the mature TRG repertoire. The biases in a given TR repertoire can stem from properties of the gene rearrangement process, as well as from thymic selection and the expansion of T cell clones. In the present work, we can make the following considerations: i) it seems to be a double preferentiality and that of the gene TRGV1 with respect to the gene TRGV2 as well as of the TRG V1-J2 rearrangement with respect to the five others (Fig. 4), the latter being supported given the comparison between the frequency of the in-frame and out-of-frame rearrangements both in cDNA and in genomic DNA (Additional file 11); ii) the fact that unrelated subjects show not only a biased usage of V-J genes, but also a biased number of nucleotides inserted/deleted at junction regions (Fig. 4 and Fig. 5c), could be explained by the presence of common antigens which can stimulate and expand T cells with a particular type of gamma chain, suggesting the existence of a basic “public” repertoire of a given TR in a range of public T cell responses; iii) finally we propose that the occurrence of clonotypes shared by different individuals who live both in marine and in artificial marine “habitat”, described as “convergent recombination” [28], could be strictly related to the biased V-J recombinational event.

The mechanisms that determine biases in genes use remain unclear. In a recent paper [36] a physical model of chromatin conformation at the TRB D-J genomic locus explains more than 80 % of the biases in TRBJ use that was measured in murine T cells. As a consequence of these structural and other biases, TR sequences are produced with different a priori frequencies, thus affecting their probability of becoming public TR that are shared among individuals. In dolphin, we could explain the abundance of TRGV1-J2 repertoire among individuals hypothizing that this combination could be produced by the rearrangement process with different a priori probabilities because an expanded role of chromatin conformation in TRGV-J rearrangement, which controls both the gene accessibility and the precise determination of gene use.

An evolutionary correlation between the dolphin TRGV1 and the human TRGV9 (Additional file 4A) genes and the dolphin TRGJ2 and the human TRGJP (Additional file 15) genes seems to exist, as in these two species the same mechanism pushes to an accurate determination of the J gene usage. In fact, dolphin TRGJ2 this work) and human TRGJP, are the most frequently used J genes in the peripheral γδ T cells [16] and occupy an intermediate position with respect to the other two J genes. At present we have knowledge of the position of the genes on the physical map for human (IMGT), dromedary [23], dolphin (this work) and sheep [9] (Additional file 1) and cattle [10] TRG loci.

It is admitted that the expressed γ/δ T cell repertoire partly depends upon preferentially rearranged TRGV-J gene combinations, indeed in human the gamma delta TCR repertoire is accompanied by selection for public gamma chain sequences such that many unrelated individuals overlap extensive in their circulating repertoire [37]. As a conseguence, the high frequency of TRGV1-J2/TRDV1-D1-J4 productive rearrangements in dolphins may represent a situation of oligoclonality comparable to that found in human with TRGV9-JP/TRDV2-D-J T cells, and in primates.

The similarity in dolphin and human of a basic public γδ repertoire, seems to be correlated with other recent findings. McGowen discovered several genes, potentially under positive selection in the dolphin lineage, associated with the nervous system, including those related to human intellectual disabilities, synaptic plasticity and sleep [38]. Moreover bottlenose dolphins are the only animals with man and apes, to be able to recognize themselves when confronted with a mirror [39], and have demonstrated the numerical skills [40]. While here, in the present work, the functional convergence of γδ domains is suggested among mammals, recently it was proposed similarity of dual-function TRA and TRD genes in jawed vertebrates and in the VLRA and VLRC genes in jawless vertebrates and their differential expression in two major T cell lineages [41–43]. Therefore comparative immunobiology of different vertebrate lineages may reveal heretofore unrealized features.

## Conclusions

The present study identifies the genomic organization and the gene content of the TRG and the TRA/TRD loci in the high quality draft sequence of the bottlenose dolphin (*Tursiops truncatus*) genome. The genomic structure of the smallest TRG locus thus described in mammals, includes two TRGV, three TRGV and only one TRGC genes. Through phylogenetic and expression analyses, 8 TRAV were assigned unambiguously to genes annotated in the TRA/TRD locus genomic sequence, while 12 TRAV could be assigned to new genes, belonging to five different subgroups. The presence of several variable genes belonging to the TRDV1 subgroup, makes the TRA/TRD dolphin locus more similar to the TRA/TRD locus of artiodactyls than to the human locus.

By comparing multiple different individuals, we provide evidence of an unusual ratio of productive/unproductive TRG transcripts and of a bias towards TRGV1-TRGJ2 rearrangements, which were dominant among the in-frame transcripts and were rare among the out-of-frame transcripts. Moreover, the cDNA analysis revealed sharing of in-frame TRG sequences within the same and in different individuals living in a controlled environment as well as in marine environment, suggesting expansion of “public” TCR by a common antigen. The selection for public gamma chain and the high frequency of TRGV1-J2/TRDV1-D1-J4 productive rearrangements in dolphins may represent a situation comparable to that found in human with TRGV9-JP/TRDV2-D-J T cells.

## Methods

### Genome and sequence analysis

The bottlenose dolphin (*Tursiops truncatus*) genome is being sequenced at ~2X coverage (BioProject: PRJNA20367) by the Human Genome Sequencing Center at the Baylor College of Medicine and the Broad Institute using a whole genome shotgun sequencing strategy [17]. In 2008, Ensembl released the first low-coverage 2.59× assembly of the dolphin (turTru1). We employed these genome assemblies using BLAST algorithm to identify the TRG and TRA/TRD loci in this species.

For the TRG locus, two overlapping scaffolds were retrieved (GEDI ID: JH473572.1; BCM-HGSC ID: contig 425448–578749), respectively of 96017 and 284974 bp (gaps included). A sequence of 188414 bp was analysed. Amphiphysin (AMPH) and related to steroidogenic acute regulatory protein D3-N-terminal like (STARD3NL), flanking TRG locus at 5′ and 3′ ends, respectively, were included in the analysis. They were predicted to be functional in dolphin (GenBank ID: XM_004317564.1; Ensembl ID: ENSTTRT00000004099). The TRG genes were identified using both our dolphin cDNA collection (this work) and the corresponding human (GEDI ID: AF159056) and sheep (GEDI ID: DQ992075.1, DQ992074.1) genomic sequences. Locations of the TRG genes are provided in Fig. 1b.

For the TRA/TRD locus, we retrieved a sequence of 482052 bp from two GenBank scaffolds, JH484271.1 and JH481615.1, and five EMBL-EBI scaffolds, Ens_742, Ens_97, Ens_89, Ens_123 and Ens_112178. Scaffold Ens_97 and Ens_123 overlap for about 16,7 Kb, including TRA14/DV4, TRA9, TRA16 and TRA17 genes, while scaffold Ens_89 and JH484271.1 overlap for about 10 Kb, a region that includes two genes, TRAV1S1 and TRAV38.1. The TRA/TRD genes were identified using the corresponding human (GEDI ID: AE000521.1) genomic sequences. Sequences of all TRA/TRD genes are in Additional file 5. Computational analysis of the dolphin TR loci was conducted using the following programs: RepeatMasker for the identification of genome-wide repeats and low complexity regions [44] (RepeatMaskerathttp://www.repeatmasker.org) and Pipmaker [45] (http://www.pipmaker.bx.psu.edu/pipmaker/) for the alignment of the dolphin sequence with the human counterpart. ClustalW (http://www.ebi.ac.uk/Tools/msa/clustalw2/) and IMGT/V-QUEST (http://www.imgt.org/IMGT_vquest/share/textes/) tools allowed the identification and characterization of the TR genes.

### Phylogenetic analyses

The TRGV, TRGJ, TRAV and TRDV genes used for the phylogenetic analyses were retrieved from IMGT/LIGM-DB and GenBank databases with the following accession numbers: AF159056 (human TRG locus), DQ992075 (sheep TRG1 locus), DQ992074 (sheep TRG2 locus), JN165102 (dromedary TRGV1), JN172913 (dromedary TRGV1), AE000521.1 (human TRA/TRD locus); sheep TRA/TRD accession numbers [14] and FN298219- FN298227 (dromedary TRD genes) [46]. Multiple alignments of the sequences under analysis were carried out with the MUSCLE program [47]. Phylogenetic analyses were performed using MEGA version 6.06 [48] and the bootstrap consensus tree inferred from 1000 replications using the Neighbor-Joining method [49, 50].

### Animals (source of tissue)

Blood samples were provided by Zoomarine Italia S.p.A. (Rome, Italy) and were collected from three dolphins, two males (Marco and King) and one female (Leah). The three individuals were born and kept under human care and are unrelated. In particular, Marco was born in the dolphinarium in Bruges (Belgium) and Tex, Marco’s father, is from the United States (Texas, Gulf of Mexico). King was born in the dolphinarium in Albufeira (Portugal), and Sam, King’s father had Cuban origins. Leah was born in the dolphinarium in Benidorm (Spain) and Eduardo, Leah’s father has Cuban origins. The identifying letters are M, K and L, respectively. The Bank for the Tissues of Mediterranean Marine Mammals (Padua, Italy) provided us a sample of skin (epidermis plus dermis) belonging to a wild dolphin, that was found beached in the Northern Adriatic Sea; for this animal the identified letter is C.

### 5′ RACE and RT-PCR

Four types of 5′ RACE and three types of RT-PCR (total of six and six experiments, respectively) on total RNA from the peripheral blood of three unrelated adult animals (identified as M, K and L) and from the skin of animal (identified as C) (Table 1 and 2) were carried out to investigate the dolphin TRG, TRA and TRD chains repertoire. Two 5′RACE experiments from the peripheral blood of the animals (identified as M and L) and three types of RT-PCR, two from blood (K and M) and one from skin (C), were carried out to investigate the dolphin TRG chain repertoire. A single 5′RACE experiment from the peripheral blood of the animal identified as L was carried out to investigate the dolphin TRA chain repertoire. Three 5′RACE experiments from the peripheral blood of the animals (identified as M and L) were carried out to investigate the dolphin TRD chain repertoire.

Total RNA was isolated from peripheral blood leukocytes (PBL) or skin using the Trizol method according to the manufacturer’s protocol (Invitrogen, Carlsbad, CA), and integrity of RNA was verified on a 1 % agarose gel. About 5 μg of total RNA were reverse transcribed with Superscript II (Invitrogen, Carlsbad, CA) by using specific primers (Table 1), designed on the sequences of the first exon for each dolphin TR constant gene sequence (TC3L for gamma chain, TA1C1L for alpha chain and TD2CL for delta chain). After linking a poly-C tail at the 5′end of the cDNAss, the cDNAds was performed with Platinum Taq Polymerase (Invitrogen) by using specific primers as lower primers, TC1L1 for gamma chain, TA1C2L for alpha chain and TD1C2L for delta chain (Table 1) and an anchor oligonucleotide as upper primer (AAP) provided from the supplier (Invitrogen). PCR conditions were the following: one cycle at 94 °C for 1 min; 35 cycles at 94 °C for 30 s, 58 °C for 45 s, 72 °C for 1 min; a final cycle of 30 min at 72 °C. The products were then amplified in a subsequent nested PCR experiment by using specific lower primers, TC1L2 for gamma chain, TA1C3L for alpha chain and TD1CL1 for delta chain (Table 1) and AUAP oligonucleotide as upper primer, provided from the supplier (Invitrogen). Nested PCR conditions were the following: one cycle at 94 °C for 1 min; 30 cycles at 94 °C for 30 s, 58 °C for 35 s, 72 °C for 30 s; a final cycle of 30 min at 72 °C.RT-PCR experiments were carried out amplifing rearranged transcripts containing TRGV1 and TRGV2 genes. Upper primers containing TRGV1 (TV1LU) and TRGV2 (TV7LU) sequences, and lower primer containing the I exon of TRGC (TC1L2) sequence were used on sscDNA (Table 1 and 2). RT-PCR conditions were: one cycle at 94 °C for 2,30 min; 35 cycles at 94 °C for 30 s, 58 °C for 40 s, 72 °C for 40 s; a final cycle of 30 min at 72 °C. The RT-PCR and RACE products were then gel-purified and cloned using StrataClone PCR Cloning Kit (Statagene). Random selected positive clones for each cloning were sequenced by a commercial service. cDNA sequence data were processed and analyzed using the Blast program (http://www.blast.ncbi.nlm.nih.gov/Blast.cgi), Clustal W2 (http://www.ebi.ac.uk/Tools/msa/clustalw2/) and IMGT_ tools (IMGT/V-QUEST) [51, 52] with integrated IMGT/JunctionAnalysis tools [53, 54] and the IMGT unique numbering for V domain [25] (http://www.imgt.org/).

### Genomic DNA isolation and PCR

Genomic DNA was extracted from whole blood of a female subject (animal identifiant letter L), with a salting-out method [55] with two modifications. First, whole blood was mixed with erythrocyte lysis buffer (155 mM NH_4_Cl, 10 mM KHCO_3_, 1 mM EDTA, pH 7.4) before the harvested white cell pellet was mixed with nucleus lysis buffer as described [55]. Second, incubation with proteinase K was carried out for 2 h at 56 °C, instead of overnight at 37 °C. The quality of the genomic DNA was evaluated by agarose gel electrophoresis and concentration determined by 260 nm absorbance measurements. Genomic PCR was performed with 50 ng to 100 ng of genomic DNA as template using specific upper primers (TV1L1 and TV7LU) designed on the two TRGV (TRGV1 and TRGV2) gene sequences in combination with two lower primers (J2GL and J5BR) designed on the two TRGJ (TRGJ2 and TRGJ3) gene sequences (Table 1). Two genomic PCR were performed to amplify TRGV1-TRGJ2 and TRGV2-TRGJ3 rearrangement combinations, respectively. High-fidelity polymerase was used to minimize possible PCR errors. PCR were performed following the manifacture’s instruction for the DNA polymerase (Platinum®Taq DNA Polymerase, Life Technologies). V1-J2 genomic PCR conditions were the following: one cycle at 94 °C for 3 min; 35 cycles at 94 °C for 30 s, 62 °C for 30 s, 72 °C for 30 s; a final cycle of 30 min at 72 °C. V2-J3 genomic PCR conditions were the following: one cycle at 94 °C for 3 min; 35 cycles at 94 °C for 30 s, 60 °C for 30 s, 72 °C for 30 s; a final cycle of 30 min at 72 °C. The obtained fragments were agarose gel purified, cloned using StrataClone PCR Cloning Kit (Stratagene) and sequenced by a commercial service. Two genomic PCR were performed to amplify TRDV4 gene using a pair of primers designed based on the relative V-exon sequence (Table 1). PCR was performed following the manufacture’s instruction for the MyTaq™ HS DNA Polymerase, (Bioline). The following settings were used: 94 °C for 2 min, followed by 30 cycles each comprising a denaturation step at 94 °C for 30 s, an annealing step of 30 s at 55 °C (according to the melting temperature of the primers), an extension step at 72 °C for 30 s, and a final extension period of 7 min at 72 °C.

### Statistical analysis

Statistical analyses were performed using 2 × 2 contingency tables. All the *p*-values shown in the Results were obtained using the Chi-squared test, considering as statistically significant a *p*- value <0.05. Fisher’s Exact test was used to confirm the significance of the Chi-squared test when the counts of observed samples had values <5. When performing multiple comparisons among in-frame and out-of-frame TRG cDNA (Additional file 12), the Chi-squared test *p*-values were adjusted using Benjamini–Hochberg false discovery rate [56]. All the analyses were performed using the R software environment for statistical computing (https://www.r-project.org/).

### Global alignments in protein secondary structure prediction and 3D visualization

Global alignment of the target and template sequences was performed with ClustalW (http://www.ebi.ac.uk/clustalw/index.html) [57]. Furthermore, when necessary, alignment was manually adjusted after predicting the secondary structure of the target and aligned to that of the template as derived with the DSSP program [58]. The secondary structure prediction was computed with SECPRED (http://gpcr.biocomp.unibo.it/cgi/predictors/s/pred_seccgi.cgi) and PSIPRED [59] and the target/template alignments were computed with YAP (http://gpcr.biocomp.unibo.it/cgi/predictors/alignss/alignss.cgi), that allows to align both primary and secondary structure at the same time. The template was selected from the Protein Data Bank (PDB) on the basis of sequence/function similarity with the target sequence and was the human γδ T cell receptor solved with an atomic solution of 3A°(PDB code and IMGT/3Dstructure-DB: 1hxm) [29, 30]. Target/ template alignments were then fed into Modeller version 9.8 [60]. For a given alignment, 50 3D models were routinely built and, then, evaluated and validated with the PROCHECK [61] and PROSA2003 [62] suites of programs. Models with the best stereochemical and energetics features were retained. 3D visualization (Additional file 14) of the RTV1M1/5R1D8 and of 5RV1M1/5R1D15 clones was computed, adopting as template the human γδ T cell receptor. The solvent accessibility was computed with DSSP program [58]. The protein complex interface were computed by the online tool PDBePISA at the EBI server (http://www.ebi.ac.uk/msd-srv/prot_int/) and visualized by UCSF Chimera tool (http://www.cgl.ucsf.edu/chimera/). The IMGT Collier de Perles of RTV1M1, 5R1D8, 5RV1M1 and 5R1D15 cDNA clonotypes were obtained using the IMGT/Collier-de-Perles tool (http://www.imgt.org) [63], starting from amino acid sequences.

## Abbreviations

CDR, complementarity determining region; FR, framework region; IG, immunoglobulins; T cell receptor gamma locus; TR, T cell receptor; TRA/TRD locus, T cell receptor alpha/delta locus; TRG locus, TRGC, T cell receptor gamma constant; TRGJ, T cell receptor gamma joining; TRGV, T cell receptor gamma variable. All TR genes (functional, ORF, pseudogenes) reported here have been approved by the IMGT/WHO- IUIS nomenclature committee and their designations are in accord with the IMGT nomenclature for human (IMGT®, the international ImMunoGeneTics information system®, http://www.imgt.org)

## Additional files

Additional file 1: Schematic representation of the genomic organization of human, sheep, dromedary and dolphin TRG loci. The diagram shows the position of all V, J, and C TRG genes according to IMGT nomenclature (http://www.imgt.org). Boxes representing genes are not to scale. Exons are not shown. (PPT 146 kb) Additional file 2: Nucleotide sequences of the dolphin TRGV (A), TRGJ (B) and TRGC (C) genes, as deduced from the genome assembly Ttru_1.4. (DOC 19 kb) Additional file 3: Dotplot matrix of dolphin/sheep (A) and of dolphin/human (B) TRG loci genomic comparison. Using the PipMaker program dolphin TRG has been plotted against sheep TRG1 (A) and dolphin TRG locus has been plotted against human (B). The transcriptional orientation of each gene is indicated by arrows and arrowheads. Dolphin TRGV1 and TRGV2 genes were classified as orthologues to their corresponding human TRGV9 gene and sheep TRGV11-1 (a pseudogene) and human TRGV11 (an ORF) and sheep TRGV7 gene, respectively (red boxes). The correspondence is due to the highest nucleotide identity (see also Additional file 4A). (PPT 363 kb) Additional file 4: The NJ tree inferred from the dolphin, sheep, dromedary and human TRGV (A) and TRGC (B) gene sequences. The evolutionary analysis was conducted in MEGA6.06 [48]. The percentage of replicate trees in which the associated taxa clustered together in the bootstrap test (1,000 replicates) is shown next to the branches [49]. The trees are drawn to scale, with branch lengths in the same units as those of the evolutionary distances used to infer the phylogenetic trees. The evolutionary distances were computed using the *p*-distance method [50] and are in the units of the number of base differences per site. (PDF 67 kb) Additional file 5: Description of the TRA/TRD genes in the dolphin genome assembly. The position of all genes and their classification and functionality are reported. (DOCX 149 kb) Additional file 6: Nucleotide (A) and deduced amino acid (B) sequences of the dolphin TRDV4 gene. TRDV4 indicates the assembly gene; G1TRDV4 and G2TRDV4 genes refer to two different individual genomic sequences. The underlined A in G2 TRDV4 may represent an allele of the TRDV4 gene. (DOCX 18 kb) Additional file 7: The NJ tree inferred from the dolphin, sheep, and human TRAV (A) and from the dolphin, sheep, dromedary and human TRDV (B) gene sequences. The evolutionary analysis was conducted in MEGA6.06 [48]. The percentage of replicate trees in which the associated taxa together in the bootstrap test (1,000 replicates) is shown next to the branches [49]. The trees are drawn to scale, with branch lengths in the same units as those of the evolutionary distances used to infer the phylogenetic trees. The evolutionary distances were computed using the *p*-distance method [50] and are in the units of the number of base differences per site. The functionality of all genes is also indicated. (A) Subgroups TRAV45 and TRAV42 are officially adopted for dolphin: these genes are related to the TRAV18 and TRAV8 subgroups, respectively. (B) Dolphin TRDV1, TRDV2 and TRDV4 gene subgroups are indicated by A, B and C, respectively. (PPT 244 kb) Additional file 8: Nucleotide and deduced amino acid sequences of the dolphin TRDJ (A) and TRDD (B) genes. The consensus sequences of the heptamer and nonamer [64] are provided at the top of the figure and underlined. The numbering adopted for the gene classification is reported on the left of each gene. The donor splice site for each TRDJ is shown. The canonical FGXG amino acid motifs are underlined. (DOC 16 kb) Additional file 9: Nucleotide and deduced amino acid sequences of the dolphin TRAJ genes. The consensus sequence of the heptamer and nonamer [64] are provided at the top of the figure and are underlined. The numbering adopted for the gene classification, is reported on the left of each gene. The donor splice site for each TRAJ is shown. The canonical FGXG amino acid motifs are underlined. (DOCX 53 kb) Additional file 10: IMGT Protein display of the TRG cDNA clones. The TRGV and TRGJ genes are listed respectively at the left and the right of the figure. Leader region (L-Region), complementarity determining regions (CDR-IMGT) and framework regions (FR-IMGT) are also indicated, according to the IMGT unique numbering for V-REGION [25]. The name of the clones are also reported. Shared sequences both within the same and in different individuals are in bold. The TRGV allele amino acid changes, if any, are green boxed. (DOC 60 kb) Additional file 11: Percentages of in-frame and out-of-frame rearranged TRG V-J cDNA (A) and percentages of in-frame and out-of-frame rearranged TRG V-J genomic DNA (B). L is animal identifiant letter. Blue areas of the bars indicate in-frame while the red areas of the bars indicate out-of-frame TRG V-J rearrangements (Fig. 4 and Additional file 10). (PPTX 37 kb) Additional file 12: Summary of the results of the statistical test: Chi-squared *p*-value is confirmed with Fisher’s *p*-value. We assume as ‘null hypothesis’ that in-frame and out-of-frame cDNAs are produced with the same probability and that there is no significant difference among in-frame and out-of frame occurrences. Thus, the null hypothesis is that the rate of in-frame and out-of frame cDNAs is proportional to the totals (or, to better say, to the remaining counts), for each category. The hypothesis has been tested using Chi-squared *p*-value and (due to the low counts) confirmed with Fisher’s *p*-value. (DOC 31 kb) Additional file 13: Overview of the analysis of the putative gd domains conducted with the software PDBePISA (M&M) (http://www.ebi.ac.uk/pdbe/pisa/). For each paired domain, 20 models were generated and after validation a representative was chosen. The columns represent, respectively: the number of H bond, the name and the position of the amino acid and of the atom involved in the H bond for delta domain; the 3× indicates that the amino acid is found in the CDR3 (IMGT_Collier de Perles) [65]. The length of the hydrogen bond expressed in angstrom, the name and position of amino acids, numeration and the atom involved in the hydrogen bond of the gamma domain, follow respectively. The 3× at the end, indicates that the amino acid is found in the CDR3 of the gamma domain. The positions highlighted in yellow indicate the salt bridge (s). (PPTX 83 kb) Additional file 14: Visualization of computationally inferred interaction between V-gamma and V-delta domain cDNA clonotypes. In RTV1M1 and 5RV1M1 V-gamma domain CDR-IMGT are blue-green-green (FR in orange); in 5R1D8 and 5R1D15 V-delta domain CDR-IMGT are red-pink-violet (FR in yellow). The protein complex interface were computed by the online tool PDBePISA at the EBI server. (http://www.ebi.ac.uk/msd-srv/prot_int/) and visualized by UCSF Chimera tool (http://www.cgl.ucsf.edu/chimera/). (PPTX 762 kb) Additional file 15: The NJ tree inferred from the dolphin, sheep, dromedary and human TRGJ gene sequences. The evolutionary analysis was conducted in MEGA6.06 [48]. The percentage of replicate trees in which the associated taxa clustered together in the bootstrap test (1,000 replicates) is shown next to the branches [49]. The tree is drawn to scale, with branch lengths in the same units as those of the evolutionary distances used to infer the phylogenetic trees. The evolutionary distances were computed using the p-distance method [50] and are in the units of the number of base differences per site. The functionality of all genes is also indicated. In the three, a clear cut subdivision of J sequences into two main sets is evident: set I (C-proximal) and set II (C-distal); genes of set III, have in the physical map an intermediate position with respect to J genes of the other two sets (Additional file 1). (PPT 159 kb)
