# Erratum to: Genomic and expression analyses of *Tursiops truncatus* T cell receptor gamma (TRG) and alpha/delta (TRA/TRD) loci reveal a similar basic public γδ repertoire in dolphin and human

**DOI:** 10.1186/s12864-016-3142-z

**Published:** 2016-10-05

**Authors:** Giovanna Linguiti, Rachele Antonacci, Gianluca Tasco, Francesco Grande, Rita Casadio, Serafina Massari, Vito Castelli, Arianna Consiglio, Marie-Paule Lefranc, Salvatrice Ciccarese

**Affiliations:** 1 Department of Biology, University of Bari, via E. Orabona 4, 70125 Bari, Italy; 2 Biocomputing Group, CIRI-Health Science and Technologies/Department of Biology, University of Bologna, via Selmi 3, 40126 Bologna, Italy; 3 Zoomarine Italia SpA, via Casablanca 61, 00071 Pomezia, RM Italy; 4 Department of Biological and Environmental Science e Technologies, University of Salento, via per Monteroni, 73100 Lecce, Italy; 5 CNR, Institute for Biomedical Technologies of Bari, via Amendola, 70125 Bari, Italy; 6 IMGT®, the international ImMunoGeneTics information system®, Laboratoire d’ImmunoGénétique Moléculaire, Institut de Génétique Humaine, UPR CNRS 1142, University of Montpellier, 34396 Montpellier Cedex 5, France

## Erratum

Unfortunately in the original publication [1] the following text is missing from the Acknowledgments section: The funds of the Puglia Region are derived from the freed resources referred to P.O.R. Puglia for Scholarships for attendance of Ph.D courses activated by Apulian Universities for the XXVI Cycle.
