# Supplementary material for: Genomic and expression analyses of Tursiops truncatus T cell receptor gamma (TRG) and alpha/delta (TRA/TRD) loci reveal a similar basic public γδ repertoire in dolphin and human
Source: BMC Genomics. 2016 Aug 15;17:634. doi: 10.1186/s12864-016-2841-9 (PMC4986337; doi:10.1186/s12864-016-2841-9)
Supplement: Additional file 1: — Schematic representation of the genomic organization of human, sheep, dromedary and dolphin TRG loci. The diagram shows the position of all V, J, and C TRG genes according to IMGT nomenclature (http://www.imgt.org). Boxes representing genes are not to scale. Exons are not shown. (PPT 146 kb) [file 12864_2016_2841_MOESM1_ESM.ppt]

## Slide 1
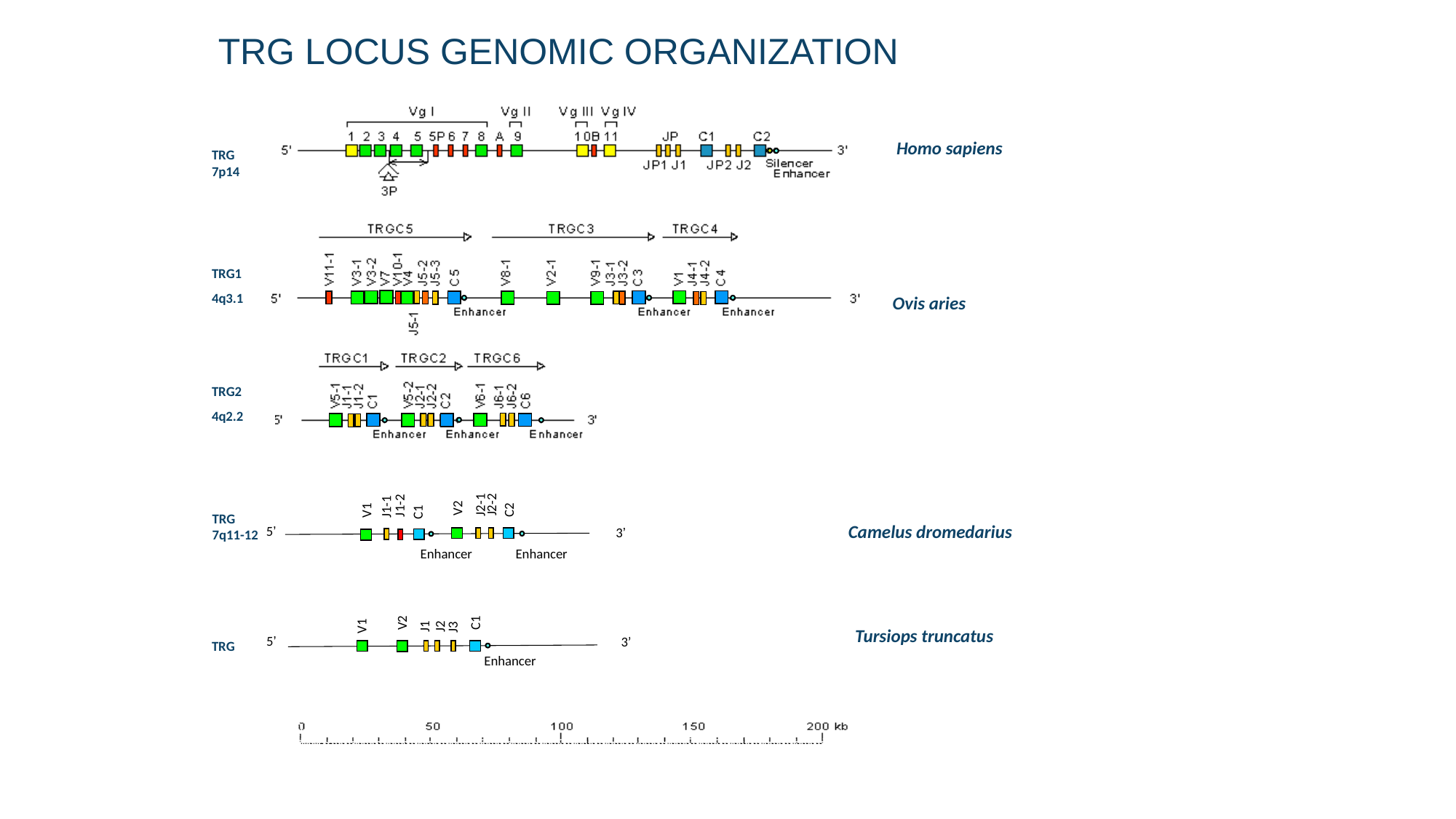

# TRG LOCUS GENOMIC ORGANIZATION
Homo sapiens
TRG 7p14
TRG1
4q3.1
Ovis aries
TRG2
4q2.2
J2-1
J1-1
V2
C2
V1
C1
5’
3’
J2-2
J1-2
TRG
7q11-12
Camelus dromedarius
Enhancer
Enhancer
C1
V2
J3
V1
J1
J2
3’
5’
Tursiops truncatus
TRG
Enhancer
