# Supplementary material for: Genomic and expression analyses of Tursiops truncatus T cell receptor gamma (TRG) and alpha/delta (TRA/TRD) loci reveal a similar basic public γδ repertoire in dolphin and human
Source: BMC Genomics. 2016 Aug 15;17:634. doi: 10.1186/s12864-016-2841-9 (PMC4986337; doi:10.1186/s12864-016-2841-9)
Supplement: Additional file 2: — Nucleotide sequences of the dolphin TRGV (A), TRGJ (B) and TRGC (C) genes, as deduced from the genome assembly Ttru_1.4. (DOC 19 kb) [file 12864_2016_2841_MOESM2_ESM.doc]

**A**

L-REGION FR1-IMGT CDR1-IMGT FR2-IMGT CDR2-IMGT FR3-IMGT CDR3-IMGT

(1-26) (27-38) (39-55) (56-65) (66-104) (105-117)

A B BC C C’ C’C’’ C’’ D E F FG

Gene (1-15) (16-26) (27-38) (39-46) (47-55) (56-65) (66-74) (75-84) (85-96) (97-104)

———————————>————————> —————> ——————> ——————> ———————> ————————> —————>

1 10 1516 23 26 27 38 3941 46 47 55 56 65 66 74 75 80 84 85 89 96 97 104 105 117

|........|....| |......|..| |..........| |.|....| |.......| |........| |.......| |....|...| |...|......| |......| |...........|

TRGV1*01 MPSLFHVLALTVLGA AGHLEQPQLSTTKKL SKTARLE**C**VVS GVTI....SKMS VYWYQERP GEAMQHLLH ILPN.NTVRR DSDVTLGKF EADKKPE..T STST**L**TIHNVQE QDAATYY**C** ALWEV........ [8.9.5]

TRGV2*01 MALLEAILFSSLWA QLKSEQPVISVTGVR DKSVVIS**C**KVS SQD.....FNEY IHWYRHKP NQGIEHLAY VVTV..PALK DLGGKKNKI EARKDVR..T STST**L**NIRFLQK EDEATYY**C** AWWV......... [7.8.4]

**B**

J-NONAMER 12–spacer J-HEPTAMER

GGTTTTTGT ************ CACTGTG

TRBJ 5'splice donor

TRGJ1 gatttttgt aggcgctctaac cattgtg ATAGGTCAGGTTGGATCAAGATATTTGGAGGAGGAACTAAGCTCATAGTAACTCTCCCTG gtaagt

R S G W I K I F G G G T K L I V T L P

TRGJ2 gctttctac aaaggcttttta gatagtg GGCTAACACATGGCAAGAGCGTCAAAGTGTTTGGTTCTGGAACACAGCTCATTGTTACAG gtaaat

L T H G K S V K V F G S G T Q L I V T

TRGJ3 agtttttga tatgggttgaat cactgtg GAGTTATTACATAAAAATTTTCGGCGATGGGACAAAACTTGTGGTCACAG gtaggt

S Y Y I K I F G D G T K L V V T

**C**

**EXTRACELLULAR-REGION**

A AB B BC C CD D DE E EF F FG G

(1-15) (16-26) (27-38) (39-45) (77-84) (85-96) (97-104) (105-117) (118-128)

——————————————> ——————————> ——————> ———————> ———————————> ———————>——————————>

1 10 15 16 2326 27 36 3941 45 77 80 84 85 89 96 97 104 105 117118 128

87654321|........|....|123|......|..| |........| |.|...|1234567|..|...|12345677654321|...|......|12|......| |...........||.......|

TRGC [EX1] (D)RSLDGDMSPKPTIFLPSIDEINL..HEAGTHL**C**LLE KFFP..DVIK VYWKEKNGNKVL..ESQQGNIIKTN.......DTYMKFSWLTVTKN....SMDNELV**C** IVKHENNKRGIDQEILFPSIEN

**CONNECTING-REGION**| **TRANSMEMBRANE-REGION** |**CYTOPLASMIC-REGION**

TRGC [EX2] (K)VTTHACVKKGS [EX3] (D)SLQLQLTNTCACYTYLLLLLKSLVYFVIISFCVCRRTAVCGHGKS
