# Supplementary figures and images for: Genomic and expression analyses of Tursiops truncatus T cell receptor gamma (TRG) and alpha/delta (TRA/TRD) loci reveal a similar basic public γδ repertoire in dolphin and human
Source: BMC Genomics. 2016 Aug 15;17:634. doi: 10.1186/s12864-016-2841-9 (PMC4986337; doi:10.1186/s12864-016-2841-9)

## Slide 1
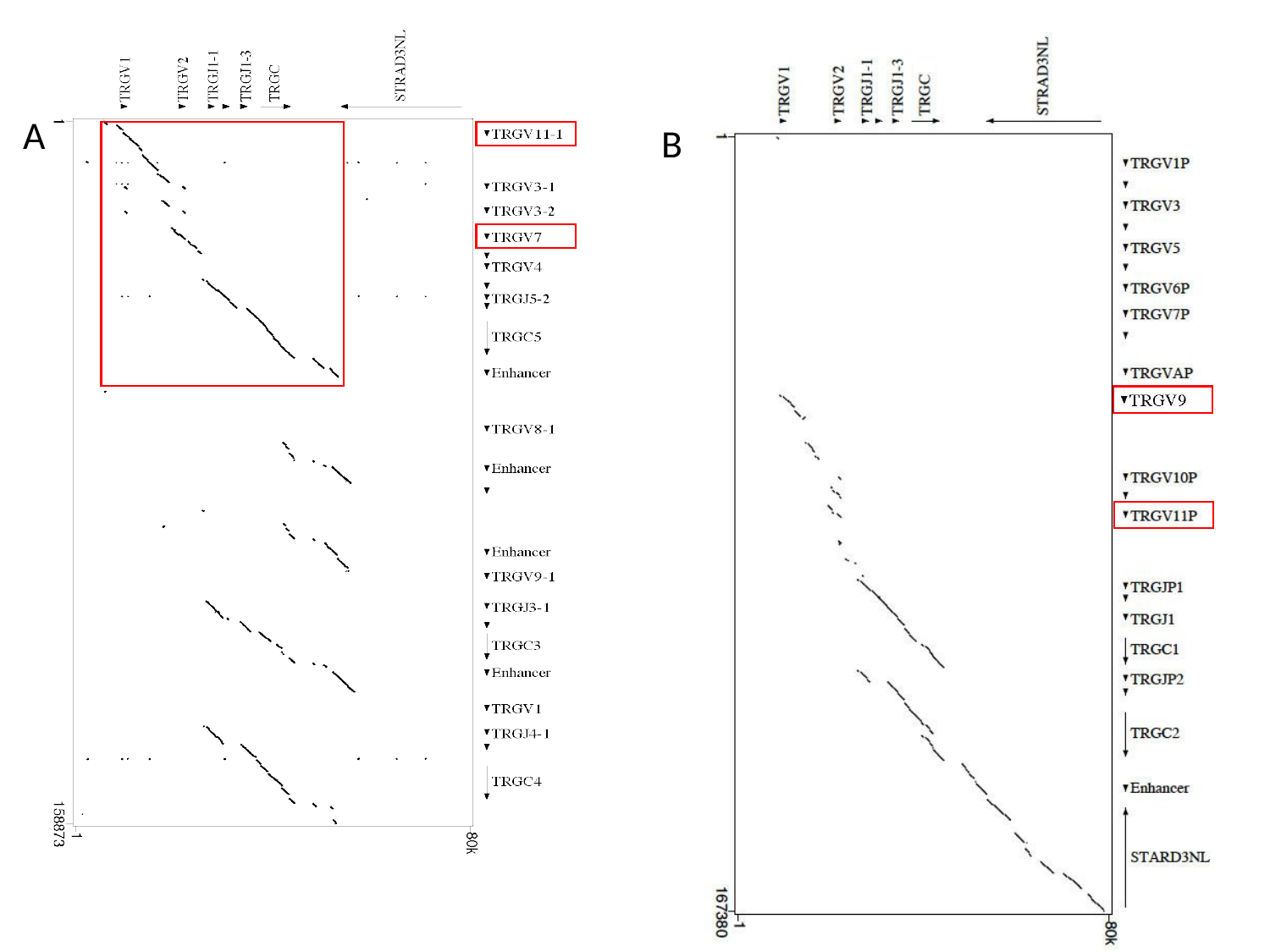

B
TRGV9
A

Supplement: Additional file 3: — Dotplot matrix of dolphin/sheep (A) and of dolphin/human (B) TRG loci genomic comparison. Using the PipMaker program dolphin TRG has been plotted against sheep TRG1 (A) and dolphin TRG locus has been plotted against human (B). The transcriptional orientation of each gene is indicated by arrows and arrowheads. Dolphin TRGV1 and TRGV2 genes were classified as orthologues to their corresponding human TRGV9 gene and sheep TRGV11-1 (a pseudogene) and human TRGV11 (an ORF) and sheep TRGV7 gene, respectively (red boxes). The correspondence is due to the highest nucleotide identity (see also Additional file 4A). (PPT 363 kb) [file 12864_2016_2841_MOESM3_ESM.ppt]

**A**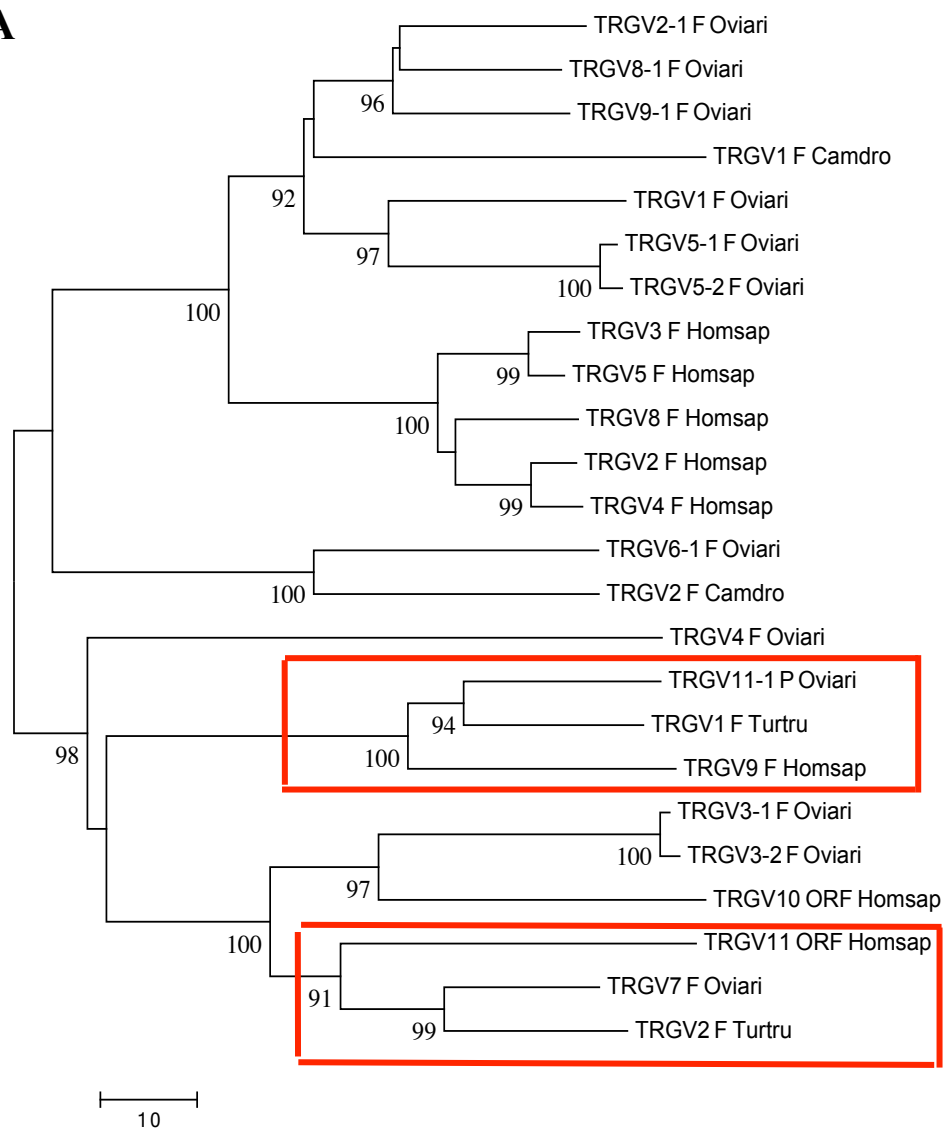

C3

C4

C1 – C2

C6

C5

C5

C5

**B**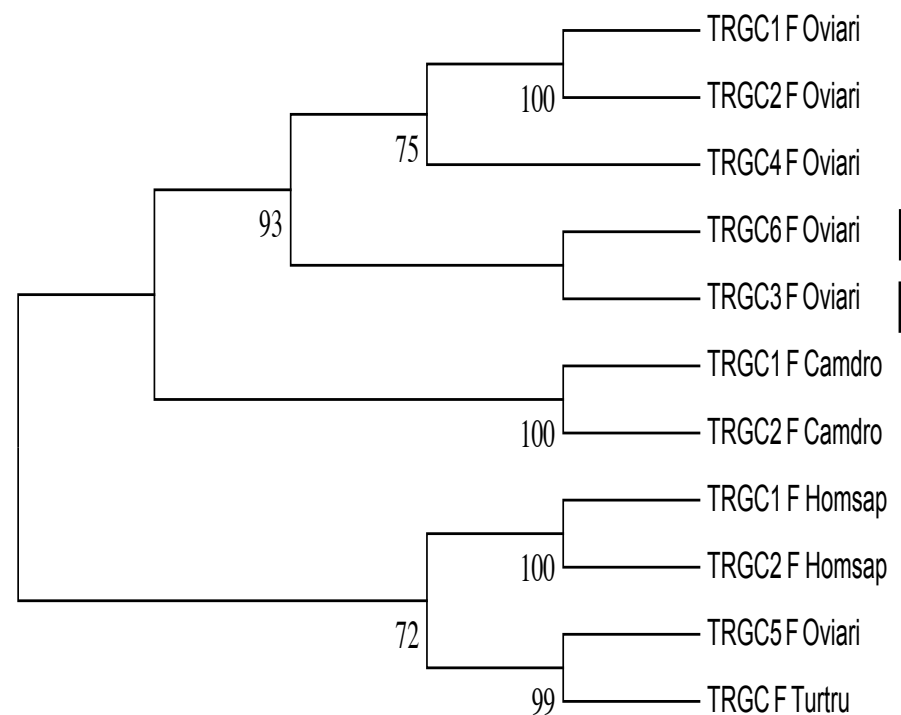

Supplement: Additional file 4: — The NJ tree inferred from the dolphin, sheep, dromedary and human TRGV (A) and TRGC (B) gene sequences. The evolutionary analysis was conducted in MEGA6.06 [48]. The percentage of replicate trees in which the associated taxa clustered together in the bootstrap test (1,000 replicates) is shown next to the branches [49]. The trees are drawn to scale, with branch lengths in the same units as those of the evolutionary distances used to infer the phylogenetic trees. The evolutionary distances were computed using the p-distance method [50] and are in the units of the number of base differences per site. (PDF 67 kb) [file 12864_2016_2841_MOESM4_ESM.pdf]

## Slide 1
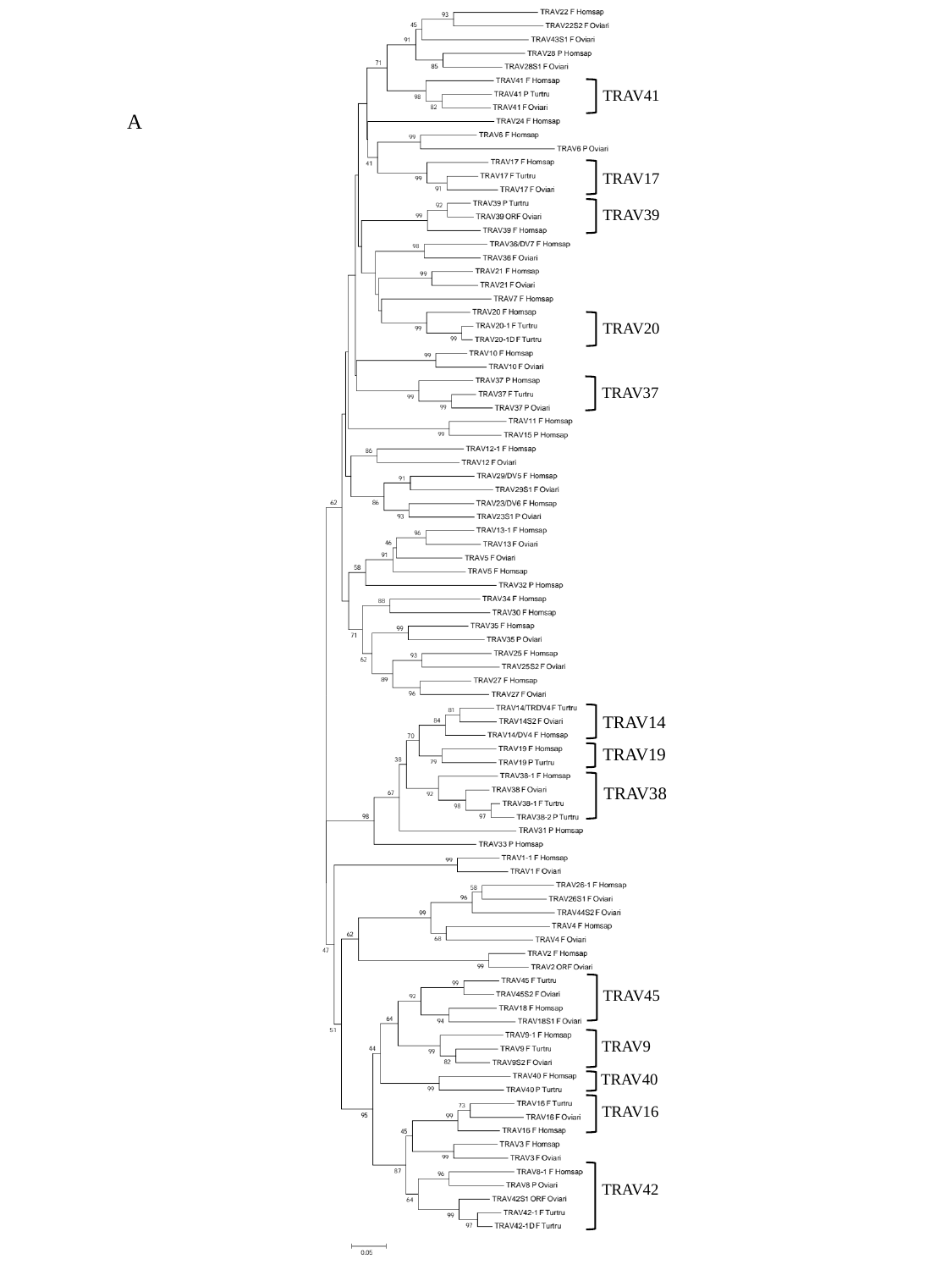

TRAV41
A
TRAV17
TRAV39
TRAV20
TRAV37
TRAV14
TRAV19
TRAV38
TRAV45
TRAV9
TRAV40
TRAV16
TRAV42

## Slide 2
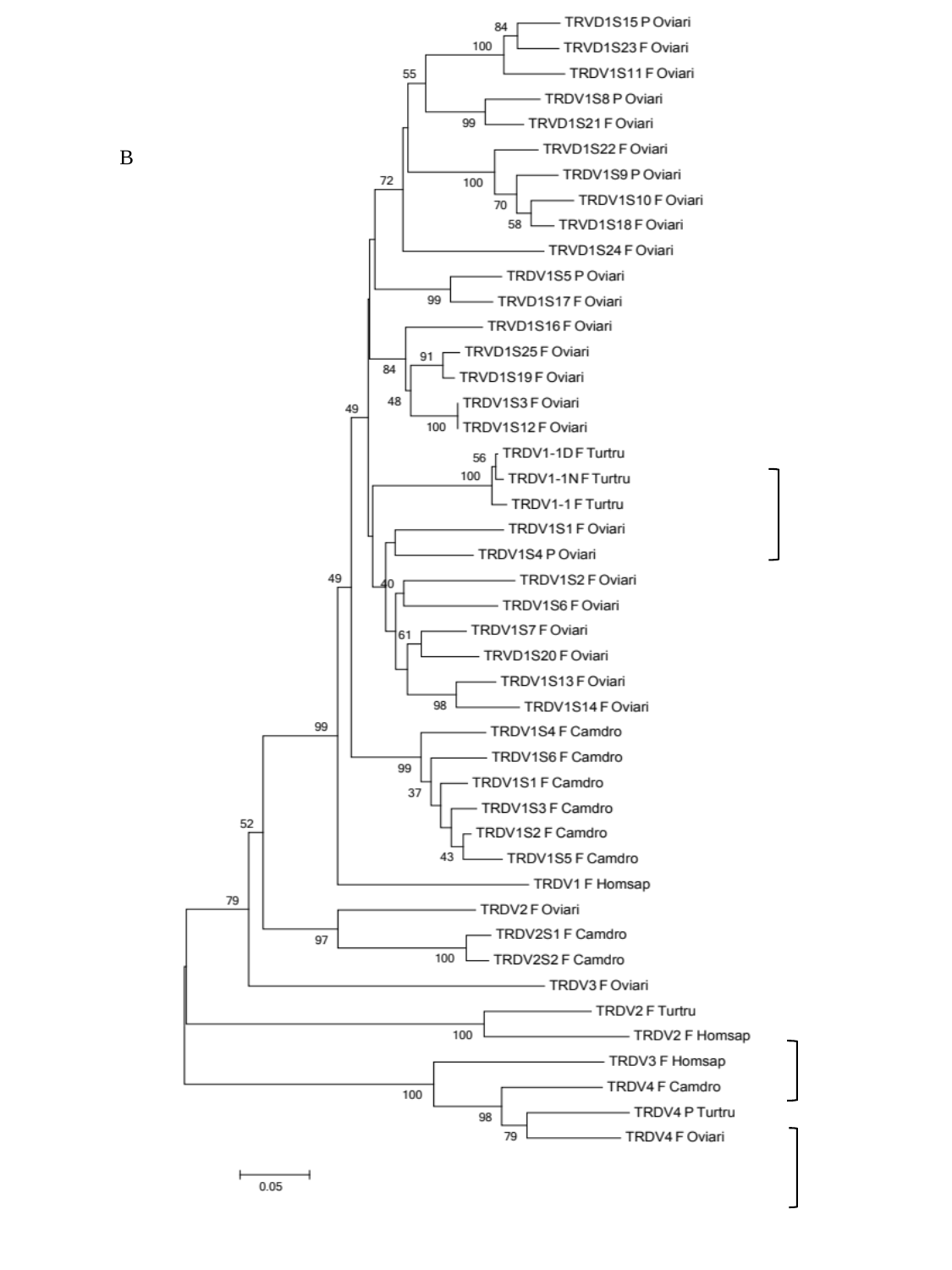

B

Supplement: Additional file 7: — The NJ tree inferred from the dolphin, sheep, and human TRAV (A) and from the dolphin, sheep, dromedary and human TRDV (B) gene sequences. The evolutionary analysis was conducted in MEGA6.06 [48]. The percentage of replicate trees in which the associated taxa together in the bootstrap test (1,000 replicates) is shown next to the branches [49]. The trees are drawn to scale, with branch lengths in the same units as those of the evolutionary distances used to infer the phylogenetic trees. The evolutionary distances were computed using the p-distance method [50] and are in the units of the number of base differences per site. The functionality of all genes is also indicated. (A) Subgroups TRAV45 and TRAV42 are officially adopted for dolphin: these genes are related to the TRAV18 and TRAV8 subgroups, respectively. (B) Dolphin TRDV1, TRDV2 and TRDV4 gene subgroups are indicated by A, B and C, respectively. (PPT 244 kb) [file 12864_2016_2841_MOESM7_ESM.ppt]

## Slide 1
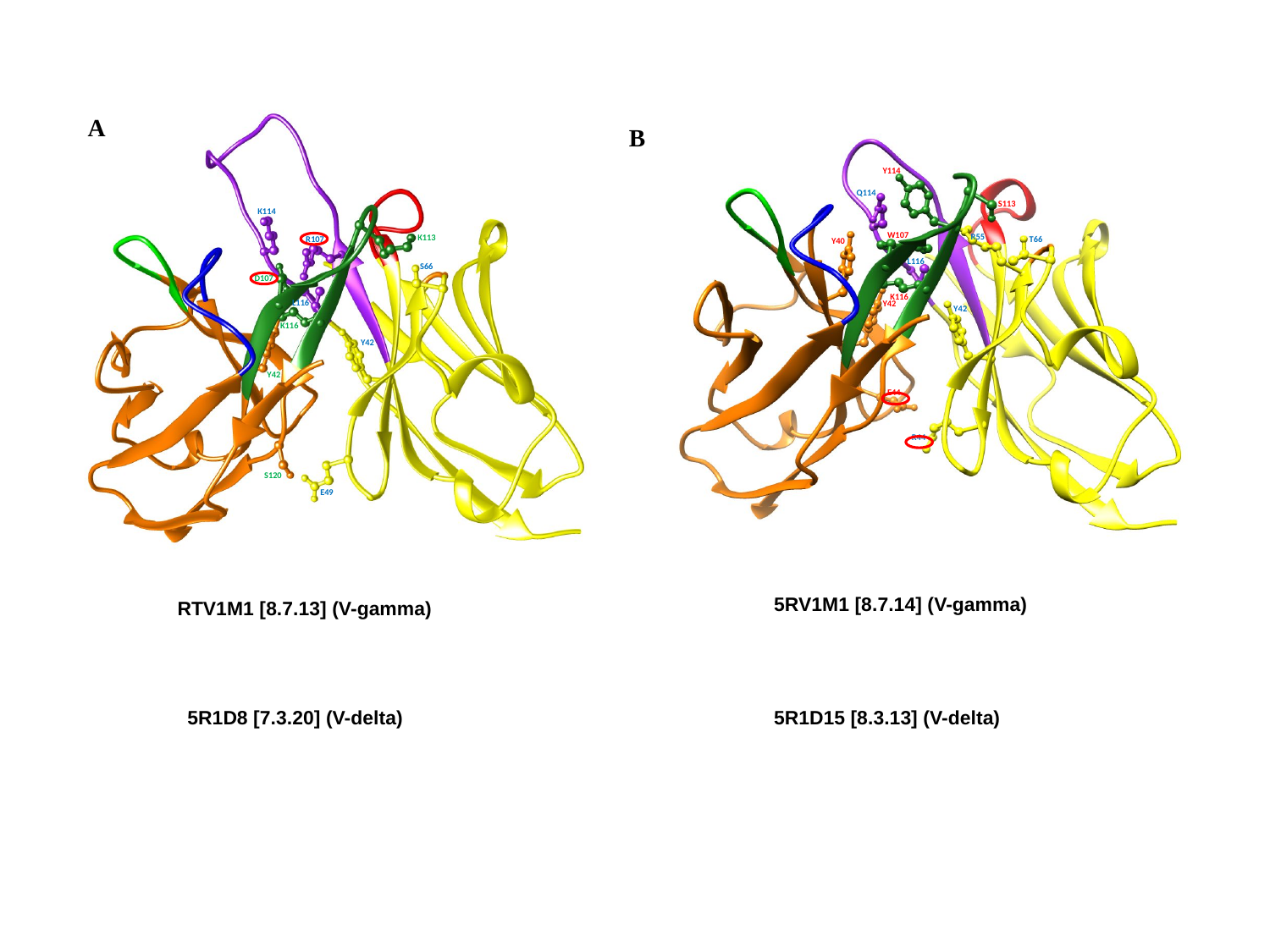

K114
K113
R107
S66
D107
L116
K116
Y42
Y42
S120
E49
RTV1M1 [8.7.13] (V-gamma)
5R1D8 [7.3.20] (V-delta)
A
B
Y114
Q114
S113
W107
R55
T66
Y40
L116
K116
Y42
Y42
E44
R44
5RV1M1 [8.7.14] (V-gamma)
5R1D15 [8.3.13] (V-delta)

Supplement: Additional file 14: — Visualization of computationally inferred interaction between V-gamma and V-delta domain cDNA clonotypes. In RTV1M1 and 5RV1M1 V-gamma domain CDR-IMGT are blue-green-green (FR in orange); in 5R1D8 and 5R1D15 V-delta domain CDR-IMGT are red-pink-violet (FR in yellow). The protein complex interface were computed by the online tool PDBePISA at the EBI server. (http://www.ebi.ac.uk/msd-srv/prot_int/) and visualized by UCSF Chimera tool (http://www.cgl.ucsf.edu/chimera/). (PPTX 762 kb) [file 12864_2016_2841_MOESM14_ESM.pptx]

## Slide 1
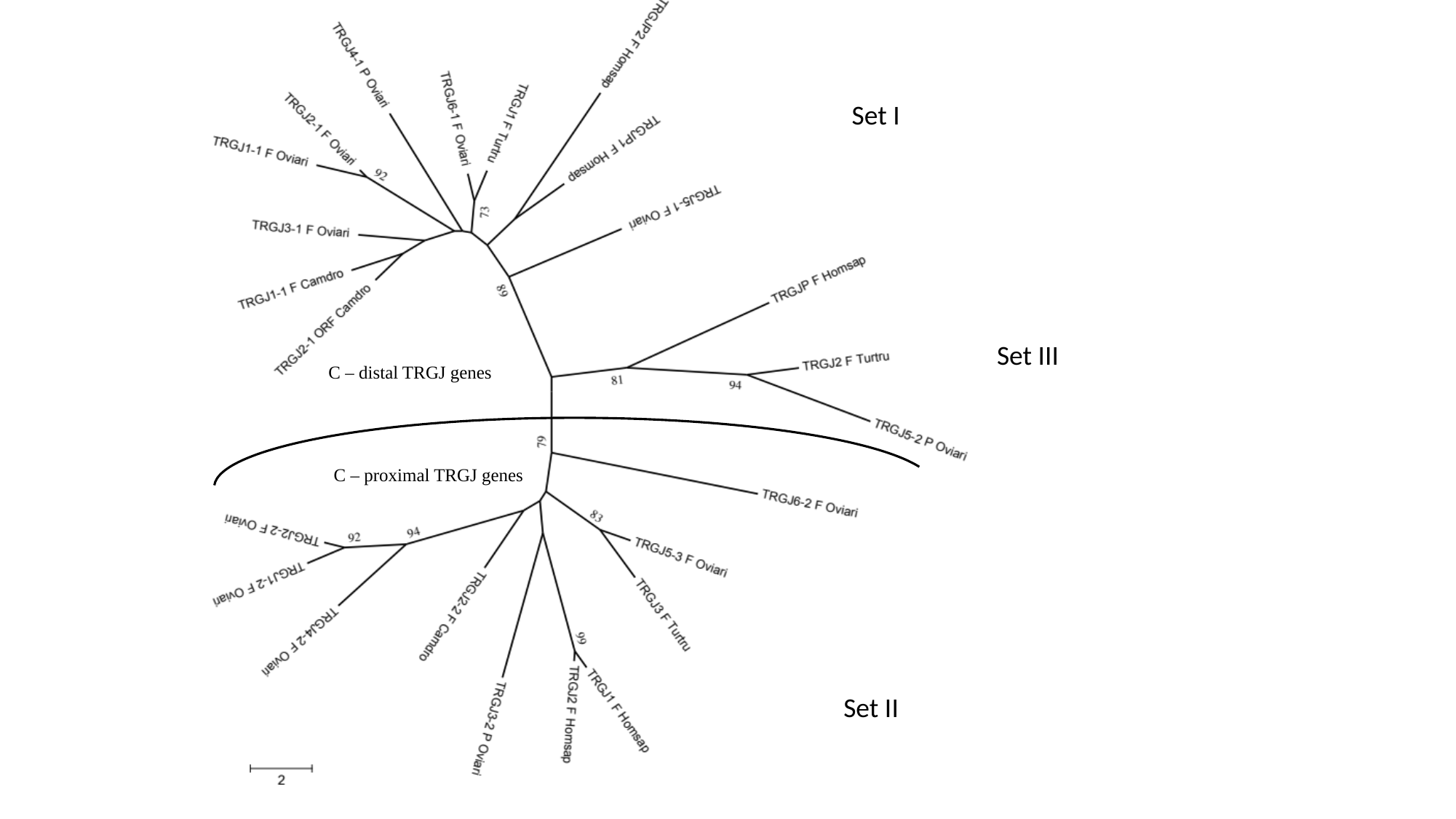

Set I
Set III
C – distal TRGJ genes
 C – proximal TRGJ genes
Set II

Supplement: Additional file 15: — The NJ tree inferred from the dolphin, sheep, dromedary and human TRGJ gene sequences. The evolutionary analysis was conducted in MEGA6.06 [48]. The percentage of replicate trees in which the associated taxa clustered together in the bootstrap test (1,000 replicates) is shown next to the branches [49]. The tree is drawn to scale, with branch lengths in the same units as those of the evolutionary distances used to infer the phylogenetic trees. The evolutionary distances were computed using the p-distance method [50] and are in the units of the number of base differences per site. The functionality of all genes is also indicated. In the three, a clear cut subdivision of J sequences into two main sets is evident: set I (C-proximal) and set II (C-distal); genes of set III, have in the physical map an intermediate position with respect to J genes of the other two sets (Additional file 1). (PPT 159 kb) [file 12864_2016_2841_MOESM15_ESM.ppt]
