# Supplementary material for: Genomic and expression analyses of Tursiops truncatus T cell receptor gamma (TRG) and alpha/delta (TRA/TRD) loci reveal a similar basic public γδ repertoire in dolphin and human
Source: BMC Genomics. 2016 Aug 15;17:634. doi: 10.1186/s12864-016-2841-9 (PMC4986337; doi:10.1186/s12864-016-2841-9)
Supplement: Additional file 5: — Description of the TRA/TRD genes in the dolphin genome assembly. The position of all genes and their classification and functionality are reported. (DOCX 149 kb) [file 12864_2016_2841_MOESM5_ESM.docx]

| Gene name | Allele name | Functionality | Positions | Lenght (nt) | Accession n° |
| --- | --- | --- | --- | --- | --- |
| TRAV20-1 | TRAV20-1*01 | F | 1723-2217 | 494 | EnsS_97 |
| TRAV8-1 | TRDV8-1*01 | F | 29964-30417 | 453 | EnsS_97 |
| TRAV14 | TRAV14*01 | F | 38669-39183 | 515 | EnsS_97 |
| TRAV9 | TRAV9*01 | F | 43523-43983 | 460 | EnsS_97 |
| TRAV16 | TRAV16*01 | F | 47564-48012 | 448 | EnsS_97 |
| TRAV17 | TRAV17*01 | F | 52973-53490 | 517 | EnsS_97 |
| TRAV18-1 | TRAV18-1*01 | F | 30741-31210 | 469 | EnsS_123 |
| TRAV19 | TRAV19*01 | P | 32484-33068 | 584 | EnsS_123 |
| TRAV20-1D | TRAV201D*01 | F | 12842-13335 | 493 | EnsS_89 |
| TRAV8-1D | TRAV8-1D*01 | F | 39421-39874 | 453 | EnsS_89 |
| TRAV37 | TRAV37*01 | F | 50197-51179 | 982 | EnsS_89 |
| TRAV38-1 | TRAV38-1*01 | F | 55034-55621 | 587 | EnsS_89 |
| TRAV38-2 | TRAV38-2*01 | P | 12287-12888 | 601 | JH484271.1 |
| TRAV39 | TRAV39*01 | P | 35000-35502 | 502 | JH484271.1 |
| TRAV40 | TRAV40*01 | P | 36267-36690 | 423 | JH484271.1 |
| TRAV41 | TRAV41*01 | P | 43975-44502 | 527 | JH484271.1 |
| TRDV1-1 | TRDV1-1*01 | F | 5496-6056 | 561 | EnsS_742 |
| TRDV1-1D | TRDV1-1D*01 | F | 19092-19651 | 559 | EnsS_97 |
| TRDV1-1N | TRDV1-1N*01 | F | 28557-29117 | 560 | EnsS_89 |
| TRDV2 | TRDV2*01 | F | 41609-42094 | 485 | JH481615.1 |
| TRDV4 | TRDV4*01 | P | 91789-92376 | 587 | JH481615.1 |
| TRDD1S1 | TRDD1S1*01 | F | 60697-60705 | 9 | JH481615.1 |
| TRDD2S1 | TRDD2S1*01 | F | 68894 -68904 | 11 | JH481615.1 |
| TRDJ1 | TRDJ1*01 | ORF | 69847-69897 | 51 | JH481615.1 |
| TRDJ4 | TRDJ4*01 | F | 75346-75394 | 49 | JH481615.1 |
| TRDJ2 | TRDJ2*01 | F | 76767-76820 | 54 | JH481615.1 |
| TRDJ3 | TRDJ3*01 | P | 83181-83239 | 59 | JH481615.1 |
|  |  |  | 85857-86136 EX1 | 280 | JH481615.1 |
| TRDC | TRDC*01 | F | 86739-86812 EX2 | 74 | JH481615.1 |
|  |  |  | 87532-87643 EX3 | 112 | JH481615.1 |
| TRAJ61 | TRAJ61*01 | F | 13685-13744 | 60 | EnsS_112178 |
| TRAJ60 | TRAJ60*01 | F | 14702-14758 | 57 | EnsS_112178 |
| TRAJ59 | TRAJ59*01 | F | 14948 -15007 | 60 | EnsS_112178 |
| TRAJ58 | TRAJ58*01 | F | 16104-16166 | 63 | EnsS_112178 |
| TRAJ57 | TRAJ57*01 | P | 17269-17330 | 62 | EnsS_112178 |
| TRAJ56 | TRAJ56*01 | P | 17910-17970 | 61 | EnsS_112178 |
| TRAJ54 | TRAJ54*01 | F | 21114-21179 | 66 | EnsS_112178 |
| TRAJ53 | TRAJ53*01 | F | 21788-21853 | 66 | EnsS_112178 |
| TRAJ52 | TRAJ52*01 | F | 24929-24990 | 62 | EnsS_112178 |
| TRAJ51 | TRAJ51*01 | F | 27522-27571 | 50 | EnsS_112178 |
| TRAJ50 | TRAJ50*01 | F | 28408-28466 | 59 | EnsS_112178 |
| TRAJ49 | TRAJ49*01 | F | 29220-29279 | 60 | EnsS_112178 |
| TRAJ48 | TRAJ48*01 | F | 31188-31244 | 57 | EnsS_112178 |
| TRAJ47 | TRAJ47*01 | F | 31736-31798 | 63 | EnsS_112178 |
| TRAJ46 | TRAJ46*01 | F | 32160-32224 | 65 | EnsS_112178 |
| TRAJ45 | TRAJ45*01 | F | 33024-33086 | 62 | EnsS_112178 |
| TRAJ44 | TRAJ44*01 | F | 34117-34173 | 57 | EnsS_112178 |
| TRAJ43 | TRAJ43*01 | F | 34918-34983 | 66 | EnsS_112178 |
| TRAJ42 | TRAJ42*01 | F | 35436-35490 | 55 | EnsS_112178 |
| TRAJ41 | TRAJ41*01 | F | 37489-37545 | 57 | EnsS_112178 |
| TRAJ40 | TRAJ40*01 | P | 39620-39683 | 64 | EnsS_112178 |
| TRAJ39 | TRAJ39*01 | P | 40263-40324 | 62 | EnsS_112178 |
| TRAJ38 | TRAJ38*01 | F | 41755-41814 | 60 | EnsS_112178 |
| TRAJ37 | TRAJ37*01 | F | 42477-42537 | 61 | EnsS_112178 |
| TRAJ36 | TRAJ36*01 | F | 44100-44162 | 63 | EnsS_112178 |
| TRAJ35 | TRAJ35*01 | F | 45149-45206 | 58 | EnsS_112178 |
| TRAJ34 | TRAJ34*01 | ORF | 46005-46061 | 57 | EnsS_112178 |
| TRAJ33 | TRAJ33*01 | F | 47075-47139 | 65 | EnsS_112178 |
| TRAJ32 | TRAJ32*01 | F | 48682-48738 | 57 | EnsS_112178 |
| TRAJ31 | TRAJ31*01 | F | 50536-50597 | 62 | EnsS_112178 |
| TRAJ30 | TRAJ30*01 | F | 51639-51698 | 60 | EnsS_112178 |
| TRAJ29 | TRAJ29*01 | F | 52664-52727 | 64 | EnsS_112178 |
| TRAJ26 | TRAJ26*01 | F | 55914-55973 | 60 | EnsS_112178 |
| TRAJ25 | TRAJ25*01 | ORF | 56219-56278 | 60 | EnsS_112178 |
| TRAJ24 | TRAJ24*01 | F | 57294-57356 | 63 | EnsS_112178 |
| TRAJ23 | TRAJ23*01 | F | 57730-57792 | 63 | EnsS_112178 |
| TRAJ22 | TRAJ22*01 | F | 59348-59410 | 63 | EnsS_112178 |
| TRAJ21 | TRAJ21*01 | F | 60870-60922 | 53 | EnsS_112178 |
| TRAJ20 | TRAJ20*01 | F | 61575-61631 | 57 | EnsS_112178 |
| TRAJ19 | TRAJ19*01 | F | 62540-62599 | 60 | EnsS_112178 |
| TRAJ18 | TRAJ18*01 | F | 62927-62992 | 66 | EnsS_112178 |
| TRAJ17 | TRAJ17*01 | F | 64215-64277 | 63 | EnsS_112178 |
| TRAJ16 | TRAJ16*01 | F | 65633-65693 | 61 | EnsS_112178 |
| TRAJ15 | TRAJ15*01 | F | 66828-66887 | 60 | EnsS_112178 |
| TRAJ14 | TRAJ14*01 | ORF | 67521-67572 | 52 | EnsS_112178 |
| TRAJ13 | TRAJ13*01 | F | 68250-68300 | 51 | EnsS_112178 |
| TRAJ12 | TRAJ12*01 | F | 69437-69496 | 60 | EnsS_112178 |
| TRAJ11 | TRAJ11*01 | F | 70009-70068 | 60 | EnsS_112178 |
| TRAJ10 | TRAJ10*01 | F | 71036-71100 | 65 | EnsS_112178 |
| TRAJ9 | TRAJ9*01 | F | 73278-73338 | 61 | EnsS_112178 |
| TRAJ8 | TRAJ8*01 | F | 73881-73940 | 60 | EnsS_112178 |
| TRAJ7 | TRAJ7*01 | F | 75916-75971 | 56 | EnsS_112178 |
| TRAJ6 | TRAJ6*01 | F | 76575-76636 | 62 | EnsS_112178 |
| TRAJ5 | TRAJ5*01 | F | 78000-78044 | 45 | EnsS_112178 |
| TRAJ4 | TRAJ4*01 | F | 80731-80793 | 63 | EnsS_112178 |
| TRAJ3 | TRAJ3*01 | F | 81698-81760 | 63 | EnsS_112178 |
| TRAJ2 | TRAJ2*01 | F | 82245-82310 | 66 | EnsS_112178 |
| TRAJ1 | TRAJ1*01 | ORF | 83373-83434 | 62 | EnsS_112178 |
|  |  |  | 86539-86793 EX1 | 255 | EnsS_112178 |
|  |  |  | 88232-88276 EX2 | 45 | EnsS_112178 |
| TRAC | TRAC*01 | F | 89151-89258 EX3 | 108 | EnsS_112178 |
|  |  |  | 90067-90609 EX4 | 543 | EnsS_112178 |
