# Supplementary material for: Genomic and expression analyses of Tursiops truncatus T cell receptor gamma (TRG) and alpha/delta (TRA/TRD) loci reveal a similar basic public γδ repertoire in dolphin and human
Source: BMC Genomics. 2016 Aug 15;17:634. doi: 10.1186/s12864-016-2841-9 (PMC4986337; doi:10.1186/s12864-016-2841-9)
Supplement: Additional file 6: — Nucleotide (A) and deduced amino acid (B) sequences of the dolphin TRDV4 gene. TRDV4 indicates the assembly gene; G1TRDV4 and G2TRDV4 genes refer to two different individual genomic sequences. The underlined A in G2 TRDV4 may represent an allele of the TRDV4 gene. (DOCX 18 kb) [file 12864_2016_2841_MOESM6_ESM.docx]

**A**

TRDV4 TAACCAAGTGACCCAGATTTCCCAGGAGCAGATACTGGTGAGTGGCAGTGAGGTGACACT

G1TRDV4 TAACCAAGTGACCCAGATTTCCCAGGAGCAGATACTGGTGAGTGGCAGTGAGGTGACACT

G2TRDV4 TAACCAAGTGACCCAGATTTCCCAGGAGCAGATACTGGTGAGTGGCAGTGAGGTGACACT

TRDV4 ACAATGCATTTTCCAAACCACATACTTAGATCCAGTCTTATACTGGTACCAAATAAGACC

G1TRDV4 ACAATGCATTTTCCAAACCACATACTTAGATCCAGTCTTATACTGGTACCAAATAAGACC

G2TRDV4 ACAATGCATTTTCCAAACCACATACTTAGATCCAG***A***CTTATACTGGTACCAAATAAGACC

TRDV4 AGATCGTTCCTTC**tag**TTTGTCCTGCACAGGAATAACATTAAATCCCATGATGCGGATCT

G1TRDV4 AGATCGTTCCTTC**tag**TTTGTCCTGCACAGGAATAACATTAAATCCCATGATGCGGATCT

G2TRDV4 AGATCGTTCCTTC**tag**TTTGTCCTGCACAGGAATAACATTAAATCCCATGATGCGGATCT

TRDV4 TGCTCGGGGTAGATTTTCCGTGCAGCACAGCCTGACCCACAAAACCTTCCAC

G1TRDV4 TGCTCGGGGTAGATTTTCCGTGCAGCACAGCCTGACCCACAAAACCTTCCAC

G2TRDV4 TGCTCGGGGTAGATTTTCCGTGCAGCACAGCCTGACCCACAAAACCTTCCAC

**B**

TRDV4 NQVTQISQEQILVSGSEVTLQCIFQTTYLDPVLYWYQIRPDRSF-FVLHRNNIKSHDADL G1TRDV4 NQVTQISQEQILVSGSEVTLQCIFQTTYLDPVLYWYQIRPDRSF-FVLHRNNIKSHDADL G2TRDV4 NQVTQISQEQILVSGSEVTLQCIFQTTYLDP*D*LYWYQIRPDRSF-FVLHRNNIKSHDADL

TRDV4 ARGRFSVQHSLTHKTFH

G1TRDV4 ARGRFSVQHSLTHKTFH

G2TRDV4 ARGRFSVQHSLTHKTFH
