# Supplementary material for: Genomic and expression analyses of Tursiops truncatus T cell receptor gamma (TRG) and alpha/delta (TRA/TRD) loci reveal a similar basic public γδ repertoire in dolphin and human
Source: BMC Genomics. 2016 Aug 15;17:634. doi: 10.1186/s12864-016-2841-9 (PMC4986337; doi:10.1186/s12864-016-2841-9)
Supplement: Additional file 8: — Nucleotide and deduced amino acid sequences of the dolphin TRDJ (A) and TRDD (B) genes. The consensus sequences of the heptamer and nonamer [64] are provided at the top of the figure and underlined. The numbering adopted for the gene classification is reported on the left of each gene. The donor splice site for each TRDJ is shown. The canonical FGXG amino acid motifs are underlined. (DOC 16 kb) [file 12864_2016_2841_MOESM8_ESM.doc]

**A**

TRDJ J-NONAMER J-SPACER J-HEPTAMER

gene name GGTTTTTCG ************ CACTGTG

J-REGION 5’splice donor

**TRDJ1** ggtatttgg aaagccctcaag cgctgta AGAGAGACAGGCTCATCTTTAGAAAAGGAACTCGCCTGATTGTGGAACCAA gtaagt

R D R L I F R K G T R L I V E P

**TRDJ4**  agtttttag actgggttaatc agctgtg TATAAGCCACTGCTATTCGGCAAAGGAACCTATCTGAACGTTGAACCAA gtaagt

Y K P L L F G K G T Y L N V E P

**TRDJ2**  acggttttt ggtcgtgatgtc tgaaatg GTGTGCTGACAGCACAACTCACCTTTGGAAACGGGACTCAACTCATCGTGGAACCGA gtaagt

V L T A Q L T F G N G T Q L I V E P

**TRDJ3** gttacctgt gaggcagcatcg caatgtg CTCCTGGGACACCTGACAGATGTTTTTTGGAGCTGGCACCAAACTCTTCGTGGAGCCCC gtgagt

S W D T * Q M F F G A G T K L F V E P

**B**

TRDD 5’D-NONAMER 5’D-SPACER 5’D-HEPTAMER 3’D-HEPTAMER 3’D-SPACER 3’D-NONAMER

gene name GGTTTTTGT ************ CACTGTG D-REGION CACAGTG *********************** ACAAAAACC

**TRDD1S1** ggttattgt actgctgtgttt cactgtg ACTACGTAC cacagag gttgaagtatattaagcctttgt tcaaaaacc

T T Y

L R

Y V

**TRDD2S1** ggtttttat aaagctctgtag cattgtg GGTGGGATACG cacagtg atacaaaacccacagagacctgt acaaaaact

G G I

V G Y

W D T
