# Supplementary material for: Genomic and expression analyses of Tursiops truncatus T cell receptor gamma (TRG) and alpha/delta (TRA/TRD) loci reveal a similar basic public γδ repertoire in dolphin and human
Source: BMC Genomics. 2016 Aug 15;17:634. doi: 10.1186/s12864-016-2841-9 (PMC4986337; doi:10.1186/s12864-016-2841-9)
Supplement: Additional file 9: — Nucleotide and deduced amino acid sequences of the dolphin TRAJ genes. The consensus sequence of the heptamer and nonamer [64] are provided at the top of the figure and are underlined. The numbering adopted for the gene classification, is reported on the left of each gene. The donor splice site for each TRAJ is shown. The canonical FGXG amino acid motifs are underlined. (DOCX 53 kb) [file 12864_2016_2841_MOESM9_ESM.docx]

| **Gene name** | **Functionaliy** | **J-NONAMER**  **GGTTTTTGT** | **12–spacer** | **J-HEPTAMER**  **CACTGTG** | **TRAJ** | **5'splice donor** |
| --- | --- | --- | --- | --- | --- | --- |
| **TRAJ61** | F | gttttttgt | aaaggtgttcac | tcctgtg | GGAATTGGGTTAACAGGAAACTGACATTTGGAGCCAAGACTAGAGGAATCTTGAAACTCA  N W V N R K L T F G A K T R G I L K L | gtaagt |
| **TRAJ60** | F | acttttggt | aaagggcccagg | cactgtg | TGAAGAGCACTGAGAAACCCACTTTTGGGAAGGGGACTCAGTTAATTGTAAACCTGG  K S T E K P T F G K G T Q L I V N L | gtgagt |
| **TRAJ59** | F | agtttatgt | aaaggcttcagc | tcctgcg | GGAAGGAAGGAAACAGGAAATTTACACTCGGAACCAGGACTCAAGTGAGAGTGAAACTTA  K E G N R K F T L G T R T Q V R V K L | gtaagt |
| **TRAJ58** | 1 F | ggtttttgc | aaagcccctcag | cattgtg | TTCAGCAAACCAGTGGCTCTCAAATGACATTTGGGAAAGGGACTCAGCTCACAGTGCATCTTG  Q Q T S G S Q M T F G K G T Q L T V H L | gtgagt |
| **TRAJ57** | P (1) | agtatttgc | aaagcagtctgt | gagggtg | TAACTCAGGGCGGATCTGAAAGACTCTCTTTGGAAAGGGAACGAAACTGACAGTAAGCCCAT  N S G R I * K T L F G K G T K L T V S P | gtaagt |
| **TRAJ56** | P (1) | agtttttgc | agagtctcgtgt | cttcgtg | TGGTACCACAGCCACTAATAAGCTGACATTCGGAAGAGGAACGACCTGAGTGTTAGACCAG  G T T A T N K L T F G R G T T * V L D Q | gtatgt |
| **TRAJ54** | 3 F | agtttgtgt | aaagctctttgc | tgtggtg | CATCTCTGGGGAGCGCCGGCCAGAAGCTGGTATTCGGGCAAGGAACCAGCCTGACCATCAACCCCA  S L G S A G Q K L V F G Q G T S L T I N P | gtgagt |
| **TRAJ53** | F | tgtttttgt | aaagcctccgct | gactgtg | AGACTAGTGGAAGTAGCAGCTATAAAGTGACATTTGGAAAAGGAACTCTCTTAATTGTGAATCCAA  T S G S S S Y K V T F G K G T L L I V N P | gtaagt |
| **TRAJ52** | 1 F | ggttcttgc | aaaggcttccat | tgcagtg | TTACCACTGGTGGTGGTGCTGGCTATGGAAAGCTGACATTTGGACAAGGAACCACTTTGACT  T T G G G A G Y G K L T F G Q G T T L T | gtccat |
| **TRAJ51** | F | cctgtcagt | tacagtgaaggg | ttgggtg | GCTGTGTGACAGCTACAGCAAGCTGACATTTGGGCAGGGGACAAGCGTATCAGTCGTTCCAA  L C D S Y S K L T F G Q G T S V S V V P | gtaagt |
| **TRAJ50** | 1 F | ggttgttgt | taagcttcctat | cacagtg | GAACACCGGCAACCAGAACTTCTATTTTGGGAGAGGGACAAGTTTGACAGTCATTCCAA  N T G N Q N F Y F G R G T S L T V I P | gtaagt |
| **TRAJ49** | F | ggtttttgt | aatgacttagaa | cactgtg | TGTCTAACTACGGAAACAAATTAACCTTCGGGAAAGGAACTAGACTCACTGTTACACCAA  S N Y G N K L T F G K G T R L T V T P | gtaagt |
| **TRAJ48** | 1 F | tctttttgt | agaggagtttga | tgctgtg | TGAATTCTGGAAACACACAGATCTTTGGCTCAGGAACCACTTTGACAGTCAAGCCTC  N S G N T Q I F G S G T T L T V K P | gtgagt |
| **TRAJ47** | F | tgtttctgt | aaagctgctgac | aacagtg | GGAAGAGTGGCAGCGGAGACAAGCTGACTTTTGGGACTGGGACGCATTTAGCAGTGAGGCCCA  K S G S G D K L T F G T G T H L A V R P | gtaagt |
| **TRAJ46** | F | gatttatgt | aaagggttgact | cgaggtg | TGAATACGGAGGAGGTATTAACAGGCTCACCTTTGGAAAAGGGACCCAGCTCATCATCCAGCCCT  E Y G G G I N R L T F G K G T Q L I I Q P | gtaagt |
| **TRAJ45** | F | ggtttctgt | catggagcatct | cacagtg | TGCCCACGGGCAGTGGCAGGAAACTCATCTTTGGGACTGGAACGAGACTTCAGGTCACCCTTG  P T G S G R K L I F G T G T R L Q V T L | gtaggt |
| **TRAJ44** | 4 F | ggtttttgt | tagagggtgtac | tgctgtg | GCAATAATTACAATGACCTACGCTTTGGCACAGGGACCAGACTGACAGTAAAACCAA  N N Y N D L R F G T G T R L T V K P | gtaagt |
| **TRAJ43** | F | ctgattact | gtgaggccccat | atgactg | TGAATTATGGAGGCAGCCAAGAAAAGCTCACCTTTGGAAAAGGCACTACACTCCCTGTTAAACCAA  N Y G G S Q E K L T F G K G T T L P V K P | gtaagt |
| **TRAJ42** | F | gtgttttgt | ttagggaaaatg | cactgtg | GAACGCTGGGTATGCATTCACCTTTGGCAAACTCACCTCGCTGCTGGTCATACTC  N A G Y A F T F G K L T S L L V I L | agtgag |
| **TRAJ41** | 2 F | ggtttacgt | agagccacgtag | cactgtg | ACACAGAAAACTACAAATACGTCTTTGGAGCAGGCACTAGCCTGCAGGTTTTAGCAA  T E N Y K Y V F G A G T S L Q V L A | gtgagt |
| **TRAJ40** | P (2) | ggtttttgc | tgagctgaagat | caccgtg | TGAATAATAATGCAGGCAACACGCTCACAATTTGGAGGGGGAACAAGGTTACTGGTCAAACCCC  * I I M Q A T R S Q F G G G T R L L V K P | gtgagt |
| **TRAJ39** | P (1) | ggtttctgt | aaagctttctat | gactgtg | TAATATTGGCAACAACCGTAAGCTGATTTGGGGATTGGGGACAAGTGTGGCAGTAAATCCAA  I L A T T V S * F G D W G Q V W Q * I Q | gtaagt |
| **TRAJ38** | 3 F | agtttttgt | aaagtagagtat | tacagtg | TGGCTCTAGCAACACAGGAAGACTCATCTTTGGGCAGGGGACGATGTTACAAGTAAAACCAG  S S N T G R L I F G Q G T M L Q V K P | gtaggt |
| **TRAJ37** | 1 F | tggttttgt | actgggcagaaa | cagtgtg | TCAGATTCTGGGGCAAACCAGCTTGTCTTTGGGATGAGAACAAGACTCACTGTCACTCCCT  S D S G A N Q L V F G M R T R L T V T P | gtaaat |
| **TRAJ36** | ORF(3) | tctgcagtg | gaatcagccgtt | gtggaca | GACAGTCAGGCTTTGGGGGTGTACGGCGCTCTGGCCCCGGCACTCAAGTGATTGTTACACCAC  Q S G F G G V R R S G P G T Q V I V T P | gtaagt |
| **TRAJ35** | 1 F | ggtttttgt | aggcctcggtat | cactgtg | TCCTACAACACAGACAAACTCATCTTTGGGGCTGGAAGCAGATTACAAGTCTTTCCAA  S Y N T D K L I F G A G S R L Q V F P | gtaagt |
| **TRAJ34** | 2 F | ggttttggt | taaggtttttgt | ctctgtg | TGGATGGCAACTATCAGTTGATCTGGGGCTCTGGGACCAAGCTAATTATAAAGCCAG  D G N Y Q L I W G S G T K L I I K P | gtaagt |
| **TRAJ33** | F | cccctcctg | aggattctgaag | ggctgtg | TAAATTATGGGAGTGCTGCCAAGGAGCTCCTCTTTGGGACTGGCCACTGCTTTCTGTCAAGCCAA  N Y G S A A K E L L F G T G H C F L S S Q | gtacgt |
| **TRAJ32** | 1 F | gtttcagta | aaggcaggaaat | gctgtgg | GGAATAACAATGCCAGAATCGTCTTTGGAACTGGAACCCAGGTGTTGGTAAAGCCCA  N N N A R I V F G T G T Q V L V K P | gtaagt |
| **TRAJ31** | F | ggtttttgt | tgtggtcccaat | cacagtg | CGAACAAACTTCTACTAACAAAATTGTCTTTGGAAGAGGGACTCAACTTCTTGTTCTCCCCA  E Q T S T N K I V F G R G T Q L L V L P | gtaagt |
| **TRAJ30** | 3 F | agtttttgt | tatggaggcaat | cactgtg | GGGATTCAGGAAACAGGCAGCTTCTCTTCGGAAAGGGCACAAGACTGGCTGTGATTCCAA  D S G N R Q L L F G K G T R L A V I P | gtaagt |
| **TRAJ29** | 2 F | tttttgcaa | agaaaggaaact | ctgtgtg | TACTCTGGAGTTGGGGGTTACCAACTCATTTTCGGGAAGGGCACCAAACTCTTGGTCATACCAA  Y S G V G G Y Q L I F G K G T K L L V I P | gtaagt |
| **TRAJ26** | 1 F | ggtttttgt | agagcccagagg | ctctgtg | GGAATAACTATGCCCAGAGTTTGACCTTCGGTGGGGGAACCAGATTGTCTGTGCTGCCCT  N N Y A Q S L T F G G G T R L S V L P | gtaagt |
| **TRAJ25** | 1 ORF(4) | ggtttttga | tgctgagataat | cactgtg | CGCAAGGACAAGGCTTCTCCCTTGTCCTTGGGAAGGGAACAAGGCTGCTTGTCAAGCCAA  Q G Q G F S L V L G K G T R L L V K P | gtaagt |
| **TRAJ24** | F | ccattttgt | aaaggagttcgt | cacagtg | TGACAACTGACGGCTGGGGGAAATTGAATTTTGGAGCAGGGACCCGGGTTGTGGTTGCCCCAG  T T D G W G K L N F G A G T R V V V A P | gtaagc |
| **TRAJ23** | 1 F | tgtttttga | caggatacgtaa | cactgtg | TGAATTATAACCAGGGAGGAAAGCTTATCTTCAGCCAGGGAACCGAGTTATCTGTGAAGCCCA  N Y N Q G G K L I F S Q G T E L S V K P | gtaagt |
| **TRAJ22** | 2 F | ggtttttgt | tgttgggcatat | cacagtg | TTTCCTCTGGTTCAAGCTGGCAACTGACCTTTGGATCTGGGACCCAACTGACTGTTGTACCTG  S S G S S W Q L T F G S G T Q L T V V P | gtaggc |
| **TRAJ21** | F | actttttgt | aatgatagtaaa | catggtg | TACAACTCCAGATTTTTACTTCGGATCTGGGACGAAACTCAGTGTAAAGCCAA  T T P D F Y F G S G T K L S V K P | gtaagt |
| **TRAJ20** | 1 F | ggtttgcgt | aggaagacgtag | cactgtg | ACTCTAATGACTACAAGTTGACGTTTGGAGCAGGAACAACAGTAACTGTAAGAGCAA  S N D Y K L T F G A G T T V T V R A | gtaagt |
| **TRAJ19** | F | ggggtgatt | ttgcagaggaca | gaactag | TCTATCAAAGTTTTCACAAGTTCAGCTTTGGAAAGGGATCCAAACACAATGACAATCCAA  Y Q S F H K F S F G K G S K H N D N P | gtaagt |
| **TRAJ18** | F | agttcatgt | aaaggggcccag | cactgtg | TCGATAGAGACTCAGGCCTGGGGAGGCTCTACTTTGGAAAAGGAACTCAGCTAACTGTACAGCCTG  D R D S G L G R L Y F G K G T Q L T V Q P | gtgagt |
| **TRAJ17** | 1 F | ggtttttgc | tgggccccaaat | cactgtg | TGACCAACTCTGCGGGGAACAAGCTAACCTTTGGAGGAGGAACCAGGATGTTAGTCAAGCCGA  T N S A G N K L T F G G G T R M L V K P | gtgagt |
| **TRAJ16** | F | gtttttgtg | gtggaatagatc | cctgtgg | GGGTTTTCAAGCAGCCTGAAGCTGGTTTTTGGAAGGGGGACCATGTTAAAGGTGAATCTTA  G F S S S L K L V F G R G T M L K V N L | gtaagt |
| **TRAJ15** | F | ggtatttgc | agggcctcgttt | cactgtg | CCTACCAGACAGGAAATGTGTTGGTCTTTGGGAAAGGAACCACCGTATCAGTATGTTCCA  Y Q T G N V L V F G K G T T V S V C S | gtaagt |
| **TRAJ14** | ORF (5) | aattcttgt | caggcagcacgg | tgctgtg | ATTTATAATACATTCATCTATGGGACTGGGACAGGATTATCAATAAAACCCA  I Y N T F I Y G T G T G L S I K P | Gtaagt |
| **TRAJ13** | 1 F | ccattctgt | agaggcctgaaa | tgcagtg | TGAATTCTGGGGGTAACCTAAAGGTTACCTTTGGAACTGGAACAACGCTTCAAGTCACCCCAA  N S G G N L K V T F G T G T T L Q V T P | gtaagt |
| **TRAJ12** | 1 F | tgtttttgg | ctgactaagaaa | cactgtg | GGAAGGGTGGAGGCTATACGTGGATCTTTGGGAGCGGGACAAGACTGCTGGTCAGGGCTG  K G G G Y T W I F G S G T R L L V R A | gtgagt |
| **TRAJ11** | 1 F | catttttgt | atggggggttgc | catagtg | TGAACTCAGGATACGGCAAAGTTACTTTTGGAAAGGGGACTATGCTTCTGGTCTCTCCAG  N S G Y G K V T F G K G T M L L V S P | gtaaat |
| **TRAJ10** | F | agtttattg | tgaggcatggga | cactgtg | GGACTCATGGGGAGGAGCAAACAAACTCACCTTTGGGAAAGGCACCTGGCTAAAAGTGGAGCTGA  D S W G G A N K L T F G K G T W L K V E L | gtaagt |
| **TRAJ9** | F | ctattttgt | cacagcacaaat | cactgtg | GGAAATAGTGGAGGCTCCAAATTTGTCTTCGGGACAGGAACAAGGCTATTTGTTGAAGCAA  G N S G G S K F V F G T G T R L F V E A | gtaagt |
| **TRAJ8** | F | ccattttgt | atagagttatgt | cagagtg | TGAACACAGGTTATCAGAAATTCGTATTTGGAACTGGCACCCAACTTTTGATCAGCCCAA  N T G Y Q K F V F G T G T Q L L I S P | gtaagt |
| **TRAJ7** | 1 F | cctttttgt | aatgcactttcc | cagagtg | TGATTATGGGAACAGATTCACTTTTGGAAAGGGGACTCGAGTATTGGTCACACCAA  D Y G N R F T F G K G T R V L V T P | gtaagt |
| **TRAJ6** | 1 F | ggttttagc | aaaggctttcct | cgctgtg | TGTATCAGGAAGAAACTATGGACTTATATTTGGAAGAGGGACCAGGCTTGTTGTTCATCCAT  V S G R N Y G L I F G R G T R L V V H P | gtgagt |
|  |  |  |  |  |  |  |
| **TRAJ5** | F | ggtatttgt | actgcattgtac | cagggtg | TGGACACAAGCAGCAGTCACTTTCGGGAGTGGAACAAGACTCCAA  W T Q A A V T F G S G T R L Q | gtgcat |
| **TRAJ4** | F | agttcctgt | aaagcaccctcc | tagtgtg | TGTTGTCTGGTGGCTACAATAAGCTGATCTTTGGAGCGGGGACCAGGCTGACCGTACGCCCGT  L S G G Y N K L I F G A G T R L T V R P | gtgagt |
| **TRAJ3** | 2 F | aggttattg | caaagaccttac | cctcagt | GGGGATTCGGCAGTGGTAACAAGTTAAACTTTGGAGCAGGGACCAGACTAAGTGTCCAACCAA  G F G S G N K L N F G A G T R L S V Q P | gtaagt |
| **TRAJ2** | F | agtttctgt | aatggtgtcacc | tgcagtg | TGAATACCAGAGGAGTGACCGATAAACACACATTTGGGAAAGGAACGCAAGTGGCCATAATATTTG  N T R G V T D K H T F G K G T Q V A I I F | gtgagt |
| **TRAJ1** | ORF (6) | gctctctgt | aatggagacatt | cagagtg | GTATGGAGGTATTGCCTCCCAGATGCAATGTGGTAAGGGAACCAGAGTTTCCATTACTCCCG  Y G G I A S Q M Q C G K G T R V S I T P | gtaagt |
|  |  |  |  |  |  |  |

**Notes:**

(1) Pseudogene because of frameshift in J-REGION

(2) Pseudogene because the J-REGION starts with a stop codon

(3) ORF because of SER instead of J-PHE

(4) ORF because of LEU instead of J-PHE

(5) ORF because of TYR instead of J-PHE

(6) ORF because of CYS instead of J-PHE
