# Supplementary material for: Genomic and expression analyses of Tursiops truncatus T cell receptor gamma (TRG) and alpha/delta (TRA/TRD) loci reveal a similar basic public γδ repertoire in dolphin and human
Source: BMC Genomics. 2016 Aug 15;17:634. doi: 10.1186/s12864-016-2841-9 (PMC4986337; doi:10.1186/s12864-016-2841-9)
Supplement: Additional file 10: — IMGT Protein display of the TRG cDNA clones. The TRGV and TRGJ genes are listed respectively at the left and the right of the figure. Leader region (L-Region), complementarity determining regions (CDR-IMGT) and framework regions (FR-IMGT) are also indicated, according to the IMGT unique numbering for V-REGION [25]. The name of the clones are also reported. Shared sequences both within the same and in different individuals are in bold. The TRGV allele amino acid changes, if any, are green boxed. (DOC 60 kb) [file 12864_2016_2841_MOESM10_ESM.doc]

L-REGION FR1-IMGT CDR1-IMGT FR2-IMGT CDR2-IMGT FR3-IMGT CDR3-IMGT FR4-IMGT CDR-IMGT

(1-26) (27-38) (39-55) (56-65) (66-104) (105-117) (118-128) length

A B BC C C' C'C" C" D E F FG G

(1-15) (16-26) (27-38) (39-46) (47-55) (56-65) (66-74) (75-84) (85-96) (97-104) (105-117) (118-128)

Clone name TRGV gene ——————————————> ——————————> ———————> ————————> ————————> —————————> —————————🡪 ———————> ———————---> TRGJ gene

1 10 15 16 23 26 27 38 3941 46 47 55 56 65 66 74 75 84 85 89 96 97 104 105 111 112 117 118 128

|........|....|AB|......|..| |..........| |.|....| |.......| |........| |.......| |........| |...|......| |......| |.....|1234321|....| |.........|

5RV1L1 V1*01 MPSLFHVLALTVLGALCVYG AGHLEQPQLSTTKKL..SKTARLE**C**VVS GVTI....SKMS VY**W**YQERP GEAMQHLLH ILPN...NIV RRDSDVTLG KFEADKKPET STST**L**TIHNVQE QDAATYY**C** ALWEVTH.......GKSVKV FGSGTQLIVT. [8.7.13] J2*01

**5RV1L2 V1*02 MPSLFHVLALTVLGALCVYG AGHLGQPQLSTTKKL..SKTARLECVVS GVTI....SKMS VYWYQERP GEAMQHLLH ILPN...NTV RRDSDVTLG KFEADKKPET STSTLTIHNVQE QDAATYYC ALWERLT......HGKSVKV FGSGTQLIVT. [8.7.14] J2*01**

**RTV1C5 V1*01 .......LALTVLGALCVYG AGHLEQPQLSTTKKL..SKTARLECVVS GVTI....SKMS VYWYQERP GEAMQHLLH ILPN...NTV RRDSDVTLG KFEADKK*A*ET STSTLTIHNVQE QDAATYYC ALWERLT......HGKSVKV FGSGTQLIVT. [8.7.14] J2*01**

**RTV1C2 V1*01 .......LALTVLGALCVYG AGHLEQPQLSTTKKL..SKTARLECVVS GVTI....SKMS VYWYQERP GEAMQHLLH ILPN...NTV RRDSDVTLG KFEADKKPET STSTLTIHNVQE QDAATYYC ALWERLT......HGKSVKV FGSGTQLIVT. [8.7.14] J2*01**

5RV1L3 V1*01 MPSLFHVLALTVLGALCVYG AGHLEQPQLSTTKKL..SKTARLE**C**VVS GVTI....SKMS VY**W**YQERP GEAMQHMLH ILPN...NTV RRDSDVTLG KFEADKKPET SKST**L**TIHNVQE QDAATYY**C** ALFTPVT......HGKSVKV FGSGTQLIVT. [8.7.14] J2*01

**RTV1M1** **V1*01 .......LALTVLGALCVYG AGHLEQPQLSTTKKL..SKTARLECVVS GVTI....SKMS VYWYQERP GEAMQHLLH ILPN...NTV RRDSDVTLG KFEADKKPET STSTLTIHNVQE QDAATYYC ARDTLTH.......GKSVKV FGSGTQLIVT. [8.7.13] J2*01**

**RTV1C1 V1*01 .......LALTVLGALCVYG AGHLEQPQLSTTKKL..SKTARLECVVS GVTI....SKMS VYWYQERP GEAMQHLLH ILPN...NTV RRDSDVTLG KFEADKKPET STSTLTIHNVQE QDAATYYC ARDTLTH.......GKSVKV FGSGTQLIVT. [8.7.13] J2*01**

**RTV1K2 V1*01 .......LALTVLGALCVYG AGHLEQPQLSTTKKL..SKTARLECVV*P* GVTI....SKMS VYWYQERP GEAMQHLLH ILPN...NTV RRDSDVTLG KFEADKKPET STSTLTIHNVQE QDAATYYC ARDTLTH.......GKSVKV FGSGTQLIVT. [8.7.13] J2*01**

**RTV1K3 V1*01 .......LALTVLGALCVYG AGHLEQPQLSTTKKL..SKTARLECVVS GVTI....SKMS VYWYQERP GEAMQHLLH ILPN...NTV RRNSDVTLG KFEADKKPET STSTLTIHNVQE QDAATYYC ARDTLTH.......GKSVKV FGSGTQLIVT. [8.7.13] J2*01**

RTV1C3 V1*02 .......LALTVLGALCVYG AGHLGQPQLSTTKKL..SKTARLE**C**VVS GVTI....SKMS VY**W**YQERP GEAMQHLLH ILPN...NTV RRDSDVTLG KFEADKKPET STST**L**TIHNVQE QDAATYY**C** ALFTPLT......HGKSVKV FGSGTQLIVT. [8.7.14] J2*01

RTV1C4 V1*01 .......LALTVLGALCVYG AGHLEQPQLSTTKKL..SKTARLE**C**VVS GVTI....SKMS VY**W**YQERP GEAMQHLLH ILPN...NTV RRDSDVTLG KFEADKKPET STST**L**TIHNVQE QDAATYY**C** ALFTPLT......HGKSVKV FGSGTQLIVT. [8.7.14] J2*01

RTV1K1 V1*01 .......LALTVLGALCVYG AGHLEQPQLSTTKKL..SKTARLE**C**VVS GVTI....SKMS VY**W**YQERP GEAMQHLLH ILPN...NTV RRDSDVTLG KFKADKKPET STST**L**TIHNVQE QDAATYY**C** ALWEQTH.......GKSVKV FGSGTQLIVT. [8.7.13] J2*01

RTV1K4 V1*01 .......LALTVLGALCVYG AGHLEQPQLSTTKKL..SKTARLE**C**VVS GVTI....SKMS VY**W**YQERP GEAMQHLLH ILPN...NTV RRDSDVTLG KFEADKKPET STST**L**TIHNVQE QDAATYY**C** ALWEQTH.......GKSVKV FGSGTQLIVT. [8.7.13] J2*01

RTV1K6 V1*01 .......LALTVLGALCVYG AGHLEQPQLSTTKKL..SKTARLE**C**VVS GVTI....SKMS VY**W**YQERP GEAMQHLLH ILPN...NTV RRDSDVTLG KFEADKKPET STST**L**TIHNVQE QDAATYY**C** ALWEQTH.......GKSVRV FGSGTQLIVT. [8.7.13] J2*01

RTV1K5 V1*01 .......LALTVLGALCVYG AGHLEQPQLSTTKKL..SKTARLE**C**MVS GVTI....SKMS VY**W**YQERP GEAMQHLLH ILPN...NTV RRDSDVTLG KFEADKKPET *T*TST**L**TIHNVQE QDAATYY**C** ALWEDDI......RGKSVKV FGSGTQLIVT. [8.7.14] J2*01

RTV1C6 V1*02 .......LALTVLGALCVYG AGHLGQPQLSTTKKL..SKTARLE**C**VVS GVTI....SKMS VY**W**YQERP GEAMQHLLH ILPN...NTV RRDSDVTLG KFEADKKPET STST**L**TIHNVQE QDAATYY**C** ALSSGS.........GWIKI FGGGTKLIVT. [8.7.11] J1*01

**RTV1K7 V1*01 .......LALTVLGALCVYG AGHLEQPQLSTTKKL..SKTARLECVVS GVTI....SKMS VYWYQERP GEAMQHLLH ILPN...NTV RRDSDVTLG KFEADKKPET STSTLTIHNVQE QDAATYYC ALWEVQL......SGSYIKI FGDGTKLVVT. [8.7.14] J3*01**

**5RV1M1 V1*01 MPSLFHVLALTVLGALCVYG AGHLEQPQLSTTKKL..SKTARLECVVS GVTI....SKMS VYWYQERP GEAMQHLLH ILPN...NTV RRDSDVTLG KFEADKKPET STSTLTIHNVQE QDAATYYC ALWEVQL......SGSYIKI FGDGTKLVVT. [8.7.14] J3*01**

RTV1K8 V1*01 .......LALTVLGALCVYG AGHLEQPQLSTTKKL..SKTARLE**C**VVS GVTI....SKMS VY**W**YQEGP GEAMQHLLH ILPN...NTV RRDSDVTLG KFEADKKPET STST**L**TIHNVQE QDAATYY**C** ALWEG...........YIKI FGDGTKLVVT. [8.7.9] J3*01

RTV1K9 V1*01 .......LALTVLGALCVYG AGHLEQPQLSTTKKL..SKTARLE**C**VVS GVTI....SKMS VY**W**YQERP GEAMQHLLH ILPN...NTV RRDSDVTLG KFEADKKPET STST**L**TIHNVQE QDAATYY**C** APSSY...........YIKI FGDGTKLVVT. [8.7.9] J3*01

RTV1K11 V1*01 .......LALTVLGALCVYG AGHLEQPQLSTTKKL..SKTARLE**C**VVS GVTI....SKMS VY**W**YQERP GEAMQHLLH ILPN...NTV RRDSDVTLG KFEADKKPET STST**L**TIHNVQE QDAATYY**C** ALWEG...........YIKI FGDGTKLVVT. [8.7.9] J3*01

RTV1K12 V1*01 .......LALTVLGALCVYG AGHLEQPQLSTTKKL..SKTARLE**C**VVS GVTI....SKMS VY**W**YQERP GEAMQHLLH ILPN...DTV RRGSDVTLG KFEADKKPET STST**L**TIHNVQE QDAATYY**C** ALWEG...........YIKI FGDGTKLVVT. [8.7.9] J3*01

5RV2L6 V2*01 .................... ............................ ............ ........ ......... .......... ......... .......... ....**L**NIRFLQK EDEATYY**C** AWWVRVG.......GYYIKI FGDGTKLVVT. [0.0.13] J3*01

5RV2L7 V2*01 ..MALLEAILFSSLWAFGLG QLKSEQPVISVTGVR..DKSVVIS**C**EVS SQDF....SNEY IH**W**YRHKP NQGIEHLAY VVT....VPA LKDLGGKKN KIEARKDVRT STST**L**NIRFLQK EDEATYY**C** AWYE.............VEI FGDGTKLVVT. [8.6.7] J3*01

**RTV2M4 V2*01 ..........FSSLWAFGLG QLKSEQPVISVTGVR..DKSVVISCKVS SQDF....SNEY IHWYRHKP NQGIEHLAY VVT....VPA LKDLGGKKN KIEARKDVRT STSTLNIRFLQK EDEATYYC AWWVLKG......GSYYIKI FGDGTKLVVT. [8.6.14] J3*01**

**RTV2C7 V2*01 ..........FSSLWAFGLG QLKSEQPVISVTGVR..DKSVVISCKVS SQDF....SNEY IHWYRHKP NQGIEHLAY VVT....VPA LKDLGGKKN KIEARKDVRT STSTLNIRFLQK EDEATYYC AWWVLKG......GSYYIKI FGDGTKLVVT. [8.6.14] J3*01**

**RTV2C8 V2*01 ..........FSSLWAFGLG QLKSEQPVISVTEVR..DKSVVISCKVS SQDF....SNEY IHWYRHKP NQGIEHLAY VVT....VPA LKDLGGKKN KIEARKDVRT STSTLNIRFLQK EDEATYYC AWWVSSS......SSYYIKI FGDGTKLVVT. [8.6.14] J3*01**

**RTV2M5 V2*01 ..........FSSLWAFGLG QLKSEQPVISVTGVR..DKSVVISCKVS SQDF....SNEY IHWYRHKP NQGIEHLAY VVT....VPA LKDLGGKKN KIEARKDVRT STSTLNIRFLQK EDEATYYC AWWVSSS......SSYYIKI FGDGTKLVVT. [8.6.14] J3*01**

RTV2C12 V2*01 ..........FSSLWAFGLG QLKSEQPVISVTGVR..DKSVVIS**C**RVS SQDF....SNEY IH**W**YRHKP NQGIEHLAY VVT....VPA LKDLGGKKN KIEARKDVRT STST**L**NIRFLQK EDEATYY**C** AWESY...........YIKI FGDGTKLVVT. [8.6.9] J3*01

RTV2M6 V2*01 ..........FSSLWAFGLG QLKSEQPVISVTGVR..DKSVVIS**C**KVS SQDF....SNEY IH**W**YRHKP NQGIEHLAY VVT....VPA LKDLGGKKN KIEARKDVRT STST**L**NIRFLQK EDEATYY**C** AWWVRSG.......PTYIKI FGDGTKLVVT. [8.6.13] J3*01

RTV2M9 V2*01 ..........FSSLWAFGLG QLKSEQPVISVIGVR..DKSVVIS**C**KVS SQDF....SNEY IH**W**YRHKP NQGIEHLAY VVT....VPA LKDLGGKKN KIEARKDVRT STST**L**NIRFLQK EDEATYY**C** AWWVGK.........YYIKI FGDGTKLVVT. [8.6.11] J3*01

RTV2K13 V2*01 ........ILFSSLWAFGLG QLKSEQPVISVTGVR..DKSVVIS**C**KVS SQDF....SNEY IH**W**YRHKP NQDIEHLAY VVT....VPA LKDLGGKKN KIEARRDVRT STST**L**NIRFLQK EDEATYY**C** AWWVI...........YIKI FGDGTKLVVT. [8.6.9] J3*01

RTV2K15 V2*01 ........ILFSSLWAFGLG QLKSEQPVISVTGVR..DKSVVIS**C**KVS SQDF....SNEY IH**W**YRHKP NQGIEHLAY VVT....VPA LKDLGGKKN KIEARKDVRT STST**L**NIRFLQK EDEATYY**C** AWWDPS.........YYIKI FGDGTKLVVT. [8.6.11] J3*01

RTV2K21 V2*01 ........ILFSSLWAFGLG QLKSEQPVISVTGVR..DKSVVIS**C**KVS SQDF....SNEY IH**W**YRHKP NQGIEHLAY VVT....VPA LKDLGGKKN KIEARKDVRT STST**L**NIRFLQK EDEATYY**C** AWWVS..........VDIKI FGDGTKLVVT. [8.6.10] J3*01

5RV2M4 V2*01 ..MALLEAILFSSLWAFGLG QLKSEQPVISVTGVR..DKSVVIS**C**KVS SQDF....SNEY IH**W**YRHKP NQGIEHLAY VVT....VPA LKDLGGKKN KIEARKDVRT STST**L**NIRFLQK EDEATYY**C** AWCSYR........SGWIKI FGGGTKLIVT. [8.6.12] J1*01

RTV2M10 V2*01 ..........FSSLWAFGLG QLKSEQPVISVTGVR..DKSVVIS**C**KVS SQDF....SNEY IH**W**YRHKP NQGIGHLAY VVT....VPA LKDLGGKKN KIEARKDVRT STST**L**NIRFLQK EDEATYY**C** AWRNLMPY.....RSGWIKI FGGGTKLIVT. [8.6.15] J1*01

5RV2M7 V2*01 ...........................................................................................................RKDVRT STST**L**NIRFLQK EDEATYY**C** AWWVSEGH.....RSGWIKI FGGGTKLIVT. [0.0.15] J1*01

RTV2K20 V2*01 ........ILFSSLWAFGLG QLKSEQPVISVTGVR..DKSVVIS**C**KVS SQDF....SNEY IH**W**YRHKP NQGIEHLAY VVT....VPA LKDLGGKKN KIEARKDVRT STST**L**NIRFLQK EDEATYY**C** AALYRS.........GWIKI FGGGTKLIVT. [8.6.11] J1*01

RTV2M11 V2*01 ..........FSSLWAFGLG QLKSEQPVISVTGVR..DKSVVIS**C**KVS SQDF....SNEY IH**W**YRHKP NQGIEHLAY VVT....VPA LKDLGGKKN KIEARKDVRT STST**L**NIRFLQK EDEATYY**C** AWWVYLT......HGKSVKV FGSGTQLIVT. [8.6.14] J2*01
