# Supplementary material for: Genomic and expression analyses of Tursiops truncatus T cell receptor gamma (TRG) and alpha/delta (TRA/TRD) loci reveal a similar basic public γδ repertoire in dolphin and human
Source: BMC Genomics. 2016 Aug 15;17:634. doi: 10.1186/s12864-016-2841-9 (PMC4986337; doi:10.1186/s12864-016-2841-9)
Supplement: Additional file 11: — Percentages of in-frame and out-of-frame rearranged TRG V-J cDNA (A) and percentages of in-frame and out-of-frame rearranged TRG V-J genomic DNA (B). L is animal identifiant letter. Blue areas of the bars indicate in-frame while the red areas of the bars indicate out-of-frame TRG V-J rearrangements (Fig. 4 and Additional file 10). (PPTX 37 kb) [file 12864_2016_2841_MOESM11_ESM.pptx]

## Slide 1
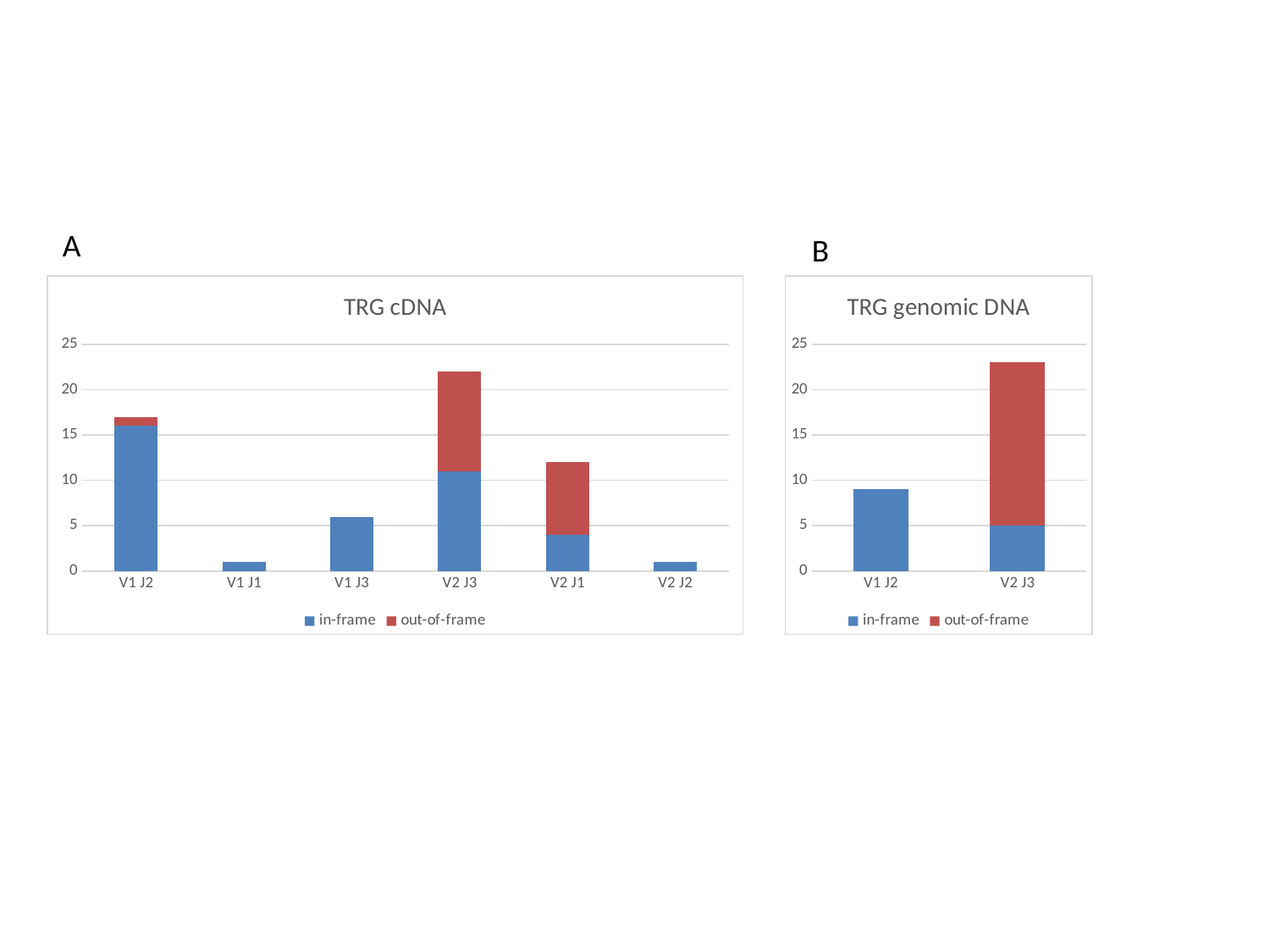

A
B
### Chart: TRG cDNA
| Category | in-frame | out-of-frame |
|---|---|---|
| V1 J2 | 16.0 | 1.0 |
| V1 J1 | 1.0 | 0.0 |
| V1 J3 | 6.0 | 0.0 |
| V2 J3 | 11.0 | 11.0 |
| V2 J1 | 4.0 | 8.0 |
| V2 J2 | 1.0 | 0.0 |
### Chart: TRG genomic DNA
| Category | in-frame | out-of-frame |
|---|---|---|
| V1 J2 | 9.0 | 0.0 |
| V2 J3 | 5.0 | 18.0 |
