# Supplementary material for: Genomic and expression analyses of Tursiops truncatus T cell receptor gamma (TRG) and alpha/delta (TRA/TRD) loci reveal a similar basic public γδ repertoire in dolphin and human
Source: BMC Genomics. 2016 Aug 15;17:634. doi: 10.1186/s12864-016-2841-9 (PMC4986337; doi:10.1186/s12864-016-2841-9)
Supplement: Additional file 12: — Summary of the results of the statistical test: Chi-squared p-value is confirmed with Fisher’s p-value. We assume as ‘null hypothesis’ that in-frame and out-of-frame cDNAs are produced with the same probability and that there is no significant difference among in-frame and out-of frame occurrences. Thus, the null hypothesis is that the rate of in-frame and out-of frame cDNAs is proportional to the totals (or, to better say, to the remaining counts), for each category. The hypothesis has been tested using Chi-squared p-value and (due to the low counts) confirmed with Fisher’s p-value. (DOC 31 kb) [file 12864_2016_2841_MOESM12_ESM.doc]

Supplementary table 1. Summary of the results of the statistical test: Chi-squared *p*-value is confirmed with Fisher’s *p*-value

| **TRG cDNA** | **In-frame** | **Out-of-frame** | **Remaining in-frame** | **Remaining out-of-frame** | **Chi-squared**  ***p*-value** | **Fisher’s**  ***p*-value** |
| --- | --- | --- | --- | --- | --- | --- |
| **V1 J2** | 16 | 1 | 23 | 19 | 0.0096 | 0.0052 |
| **V1 J1** | 1 | 0 | 39-1=38 | 20-0=20 | 1 | 1 |
| **V1 J3** | 6 | 0 | 33 | 20 | 0.1628 | 0.0867 |
| **V2 J3** | 11 | 11 | 28 | 9 | 0.0836 | 0.0528 |
| **V2 J1** | 4 | 8 | 35 | 12 | 0.019 | 0.0142 |
| **V2 J2** | 1 | 0 | 38 | 20 | 1 | 1 |
