# Supplementary material for: Genomic and expression analyses of Tursiops truncatus T cell receptor gamma (TRG) and alpha/delta (TRA/TRD) loci reveal a similar basic public γδ repertoire in dolphin and human
Source: BMC Genomics. 2016 Aug 15;17:634. doi: 10.1186/s12864-016-2841-9 (PMC4986337; doi:10.1186/s12864-016-2841-9)
Supplement: Additional file 13: — Overview of the analysis of the putative gd domains conducted with the software PDBePISA (M&M) (http://www.ebi.ac.uk/pdbe/pisa/). For each paired domain, 20 models were generated and after validation a representative was chosen. The columns represent, respectively: the number of H bond, the name and the position of the amino acid and of the atom involved in the H bond for delta domain; the 3× indicates that the amino acid is found in the CDR3 (IMGT_Collier de Perles) [65]. The length of the hydrogen bond expressed in angstrom, the name and position of amino acids, numeration and the atom involved in the hydrogen bond of the gamma domain, follow respectively. The 3× at the end, indicates that the amino acid is found in the CDR3 of the gamma domain. The positions highlighted in yellow indicate the salt bridge (s). (PPTX 83 kb) [file 12864_2016_2841_MOESM13_ESM.pptx]

## Slide 1
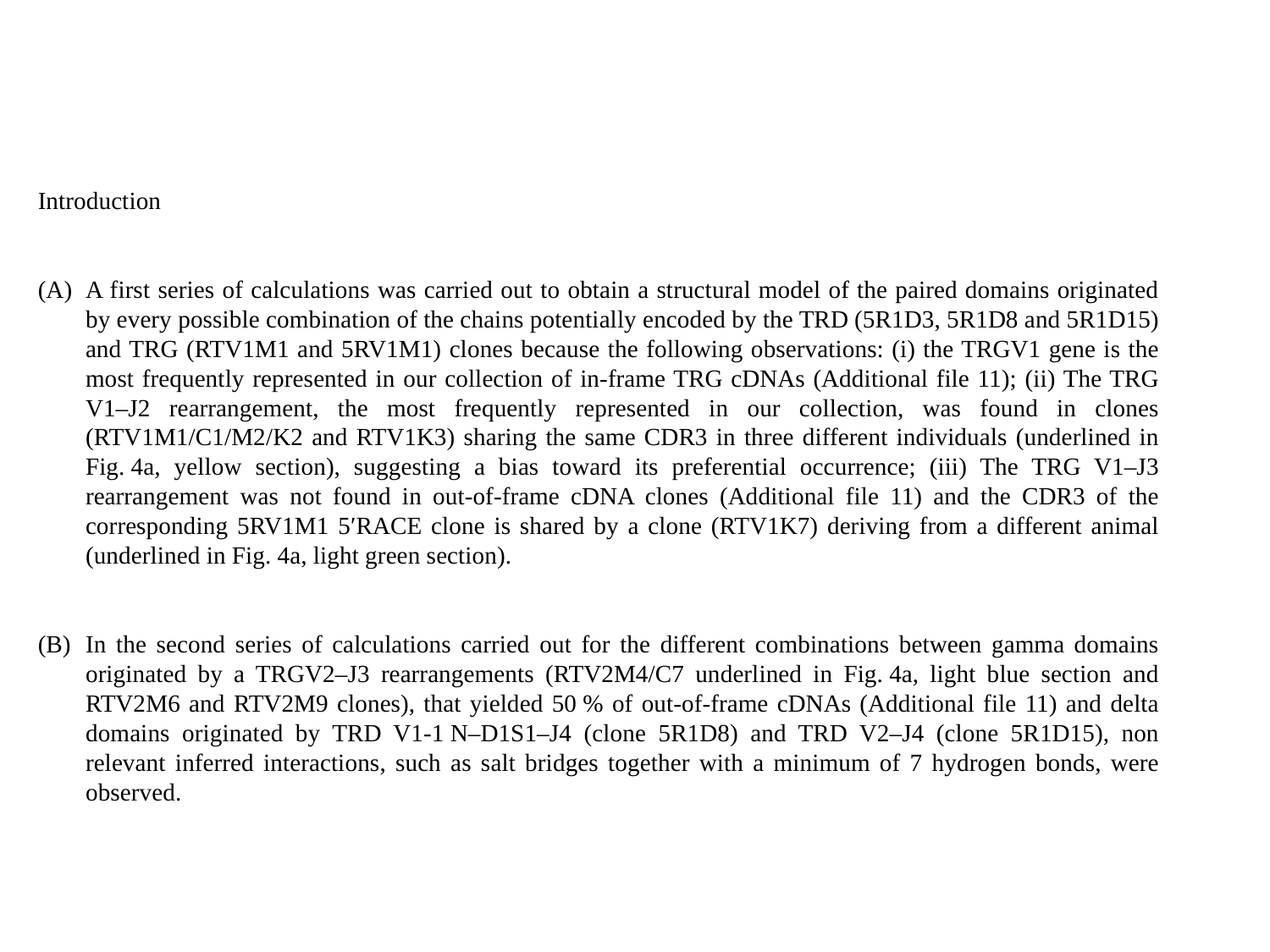

Introduction
A first series of calculations was carried out to obtain a structural model of the paired domains originated by every possible combination of the chains potentially encoded by the TRD (5R1D3, 5R1D8 and 5R1D15) and TRG (RTV1M1 and 5RV1M1) clones because the following observations: (i) the TRGV1 gene is the most frequently represented in our collection of in-frame TRG cDNAs (Additional file 11); (ii) The TRG V1–J2 rearrangement, the most frequently represented in our collection, was found in clones (RTV1M1/C1/M2/K2 and RTV1K3) sharing the same CDR3 in three different individuals (underlined in Fig. 4a, yellow section), suggesting a bias toward its preferential occurrence; (iii) The TRG V1–J3 rearrangement was not found in out-of-frame cDNA clones (Additional file 11) and the CDR3 of the corresponding 5RV1M1 5′RACE clone is shared by a clone (RTV1K7) deriving from a different animal (underlined in Fig. 4a, light green section).
In the second series of calculations carried out for the different combinations between gamma domains originated by a TRGV2–J3 rearrangements (RTV2M4/C7 underlined in Fig. 4a, light blue section and RTV2M6 and RTV2M9 clones), that yielded 50 % of out-of-frame cDNAs (Additional file 11) and delta domains originated by TRD V1-1 N–D1S1–J4 (clone 5R1D8) and TRD V2–J4 (clone 5R1D15), non relevant inferred interactions, such as salt bridges together with a minimum of 7 hydrogen bonds, were observed.

## Slide 2
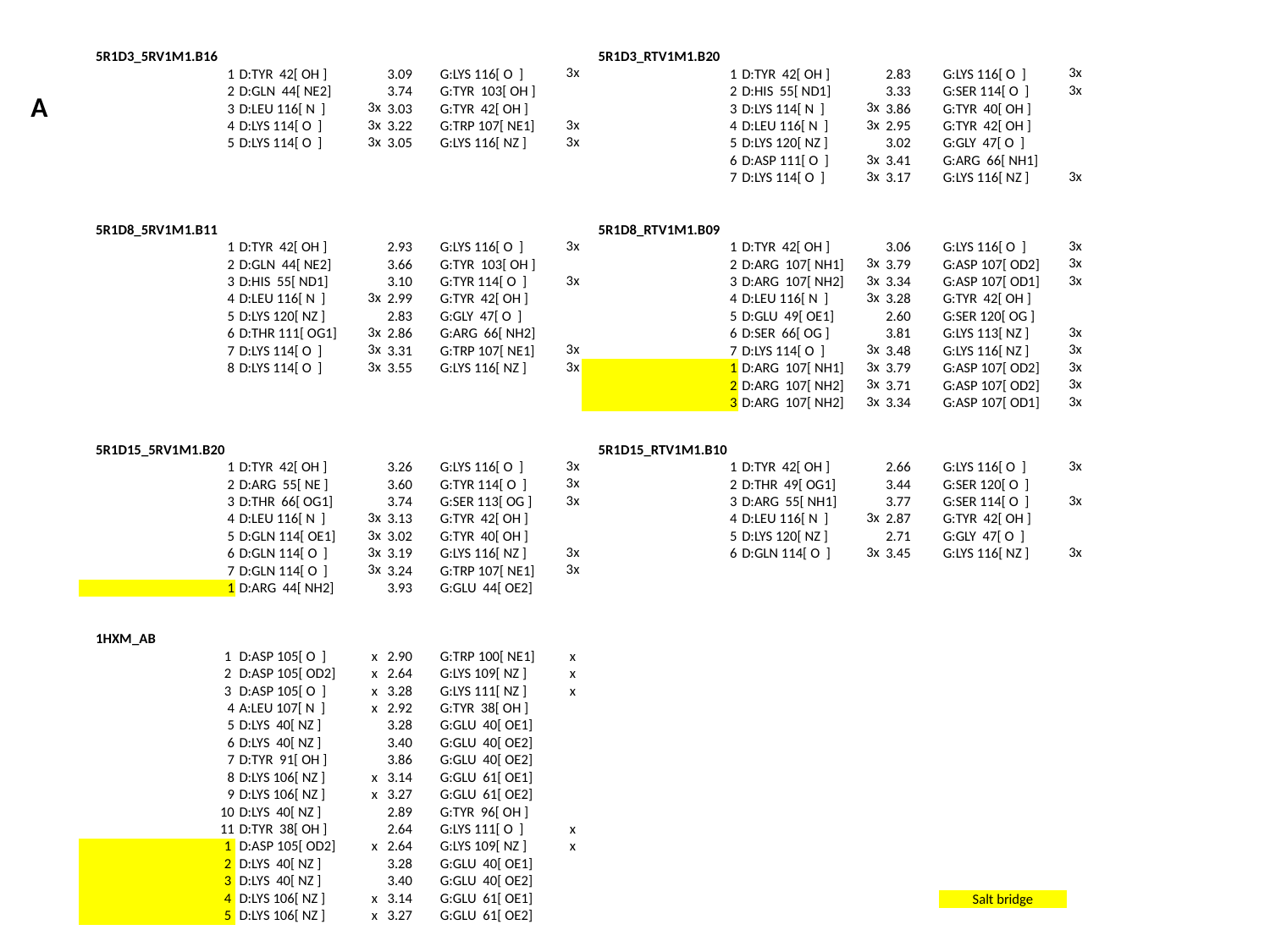

| 5R1D3\_5RV1M1.B16 | | | | | | 5R1D3\_RTV1M1.B20 | | | | | |
| --- | --- | --- | --- | --- | --- | --- | --- | --- | --- | --- | --- |
| 1 | D:TYR 42[ OH ] | | 3.09 | G:LYS 116[ O ] | 3x | 1 | D:TYR 42[ OH ] | | 2.83 | G:LYS 116[ O ] | 3x |
| 2 | D:GLN 44[ NE2] | | 3.74 | G:TYR 103[ OH ] | | 2 | D:HIS 55[ ND1] | | 3.33 | G:SER 114[ O ] | 3x |
| 3 | D:LEU 116[ N ] | 3x | 3.03 | G:TYR 42[ OH ] | | 3 | D:LYS 114[ N ] | 3x | 3.86 | G:TYR 40[ OH ] | |
| 4 | D:LYS 114[ O ] | 3x | 3.22 | G:TRP 107[ NE1] | 3x | 4 | D:LEU 116[ N ] | 3x | 2.95 | G:TYR 42[ OH ] | |
| 5 | D:LYS 114[ O ] | 3x | 3.05 | G:LYS 116[ NZ ] | 3x | 5 | D:LYS 120[ NZ ] | | 3.02 | G:GLY 47[ O ] | |
| | | | | | | 6 | D:ASP 111[ O ] | 3x | 3.41 | G:ARG 66[ NH1] | |
| | | | | | | 7 | D:LYS 114[ O ] | 3x | 3.17 | G:LYS 116[ NZ ] | 3x |
| | | | | | | | | | | | |
| | | | | | | | | | | | |
| 5R1D8\_5RV1M1.B11 | | | | | | 5R1D8\_RTV1M1.B09 | | | | | |
| 1 | D:TYR 42[ OH ] | | 2.93 | G:LYS 116[ O ] | 3x | 1 | D:TYR 42[ OH ] | | 3.06 | G:LYS 116[ O ] | 3x |
| 2 | D:GLN 44[ NE2] | | 3.66 | G:TYR 103[ OH ] | | 2 | D:ARG 107[ NH1] | 3x | 3.79 | G:ASP 107[ OD2] | 3x |
| 3 | D:HIS 55[ ND1] | | 3.10 | G:TYR 114[ O ] | 3x | 3 | D:ARG 107[ NH2] | 3x | 3.34 | G:ASP 107[ OD1] | 3x |
| 4 | D:LEU 116[ N ] | 3x | 2.99 | G:TYR 42[ OH ] | | 4 | D:LEU 116[ N ] | 3x | 3.28 | G:TYR 42[ OH ] | |
| 5 | D:LYS 120[ NZ ] | | 2.83 | G:GLY 47[ O ] | | 5 | D:GLU 49[ OE1] | | 2.60 | G:SER 120[ OG ] | |
| 6 | D:THR 111[ OG1] | 3x | 2.86 | G:ARG 66[ NH2] | | 6 | D:SER 66[ OG ] | | 3.81 | G:LYS 113[ NZ ] | 3x |
| 7 | D:LYS 114[ O ] | 3x | 3.31 | G:TRP 107[ NE1] | 3x | 7 | D:LYS 114[ O ] | 3x | 3.48 | G:LYS 116[ NZ ] | 3x |
| 8 | D:LYS 114[ O ] | 3x | 3.55 | G:LYS 116[ NZ ] | 3x | 1 | D:ARG 107[ NH1] | 3x | 3.79 | G:ASP 107[ OD2] | 3x |
| | | | | | | 2 | D:ARG 107[ NH2] | 3x | 3.71 | G:ASP 107[ OD2] | 3x |
| | | | | | | 3 | D:ARG 107[ NH2] | 3x | 3.34 | G:ASP 107[ OD1] | 3x |
| | | | | | | | | | | | |
| 5R1D15\_5RV1M1.B20 | | | | | | 5R1D15\_RTV1M1.B10 | | | | | |
| 1 | D:TYR 42[ OH ] | | 3.26 | G:LYS 116[ O ] | 3x | 1 | D:TYR 42[ OH ] | | 2.66 | G:LYS 116[ O ] | 3x |
| 2 | D:ARG 55[ NE ] | | 3.60 | G:TYR 114[ O ] | 3x | 2 | D:THR 49[ OG1] | | 3.44 | G:SER 120[ O ] | |
| 3 | D:THR 66[ OG1] | | 3.74 | G:SER 113[ OG ] | 3x | 3 | D:ARG 55[ NH1] | | 3.77 | G:SER 114[ O ] | 3x |
| 4 | D:LEU 116[ N ] | 3x | 3.13 | G:TYR 42[ OH ] | | 4 | D:LEU 116[ N ] | 3x | 2.87 | G:TYR 42[ OH ] | |
| 5 | D:GLN 114[ OE1] | 3x | 3.02 | G:TYR 40[ OH ] | | 5 | D:LYS 120[ NZ ] | | 2.71 | G:GLY 47[ O ] | |
| 6 | D:GLN 114[ O ] | 3x | 3.19 | G:LYS 116[ NZ ] | 3x | 6 | D:GLN 114[ O ] | 3x | 3.45 | G:LYS 116[ NZ ] | 3x |
| 7 | D:GLN 114[ O ] | 3x | 3.24 | G:TRP 107[ NE1] | 3x | | | | | | |
| 1 | D:ARG 44[ NH2] | | 3.93 | G:GLU 44[ OE2] | | | | | | | |
| | | | | | | | | | | | |
| | | | | | | | | | | | |
| 1HXM\_AB | | | | | | | | | | | |
| 1 | D:ASP 105[ O  ] | x | 2.90 | G:TRP 100[ NE1] | x | | | | | | |
| 2 | D:ASP 105[ OD2] | x | 2.64 | G:LYS 109[ NZ ] | x | | | | | | |
| 3 | D:ASP 105[ O  ] | x | 3.28 | G:LYS 111[ NZ ] | x | | | | | | |
| 4 | A:LEU 107[ N  ] | x | 2.92 | G:TYR  38[ OH ] | | | | | | | |
| 5 | D:LYS  40[ NZ ] | | 3.28 | G:GLU  40[ OE1] | | | | | | | |
| 6 | D:LYS  40[ NZ ] | | 3.40 | G:GLU  40[ OE2] | | | | | | | |
| 7 | D:TYR  91[ OH ] | | 3.86 | G:GLU  40[ OE2] | | | | | | | |
| 8 | D:LYS 106[ NZ ] | x | 3.14 | G:GLU  61[ OE1] | | | | | | | |
| 9 | D:LYS 106[ NZ ] | x | 3.27 | G:GLU  61[ OE2] | | | | | | | |
| 10 | D:LYS  40[ NZ ] | | 2.89 | G:TYR  96[ OH ] | | | | | | | |
| 11 | D:TYR  38[ OH ] | | 2.64 | G:LYS 111[ O  ] | x | | | | | | |
| 1 | D:ASP 105[ OD2] | x | 2.64 | G:LYS 109[ NZ ] | x | | | | | | |
| 2 | D:LYS  40[ NZ ] | | 3.28 | G:GLU  40[ OE1] | | | | | | | |
| 3 | D:LYS  40[ NZ ] | | 3.40 | G:GLU  40[ OE2] | | | | | | | |
| 4 | D:LYS 106[ NZ ] | x | 3.14 | G:GLU  61[ OE1] | | | | | | Salt bridge | |
| 5 | D:LYS 106[ NZ ] | x | 3.27 | G:GLU  61[ OE2] | | | | | | | |
A

## Slide 3
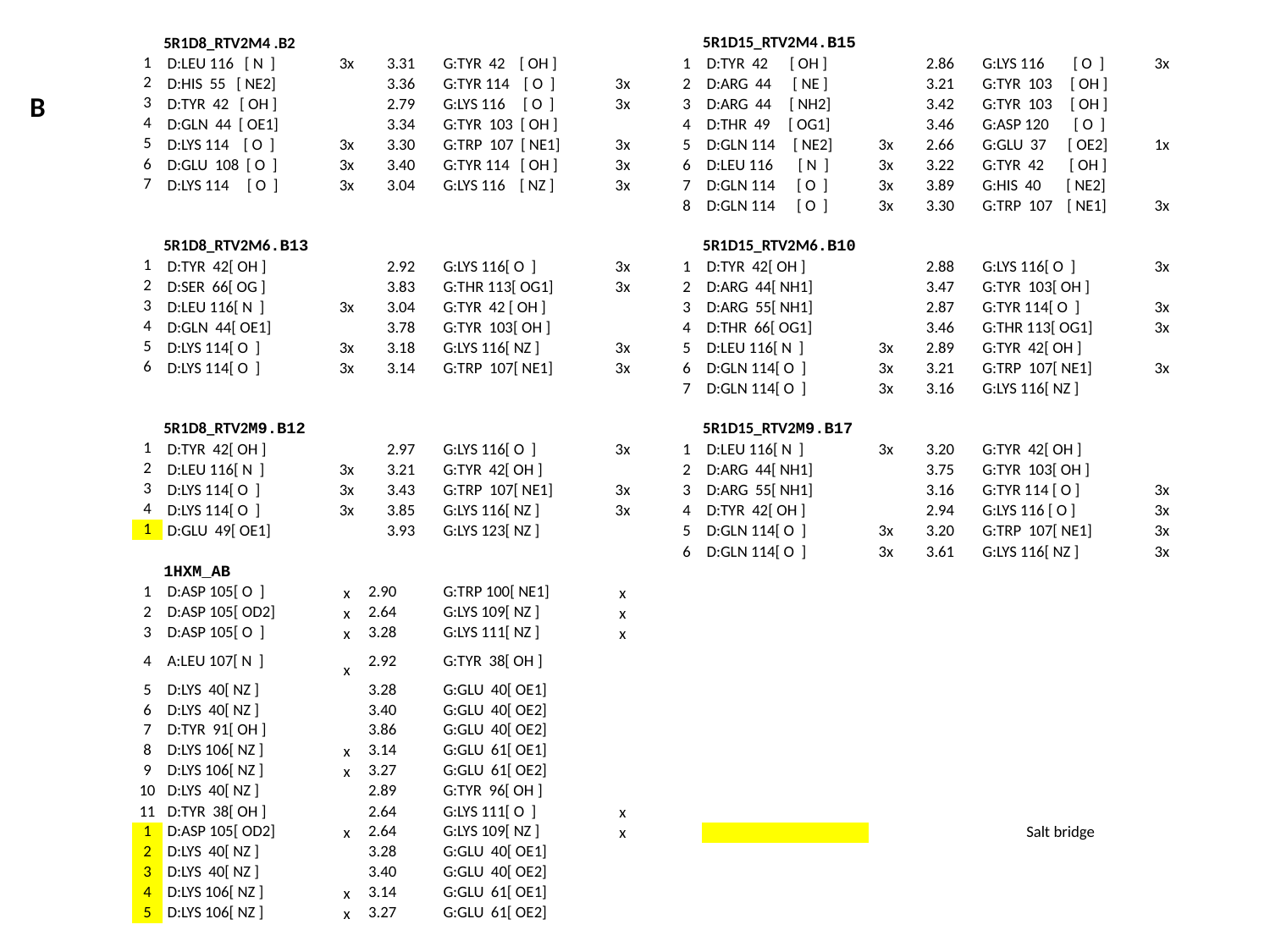

| | 5R1D8\_RTV2M4 .B2 | | | | | | | 5R1D15\_RTV2M4.B15 | | | | |
| --- | --- | --- | --- | --- | --- | --- | --- | --- | --- | --- | --- | --- |
| 1 | D:LEU 116 [ N  ] | 3x | 3.31 | G:TYR  42 [ OH ] | | | 1 | D:TYR  42 [ OH ] | | 2.86 | G:LYS 116 [ O  ] | 3x |
| 2 | D:HIS  55 [ NE2] | | 3.36 | G:TYR 114 [ O  ] | 3x | | 2 | D:ARG  44 [ NE ] | | 3.21 | G:TYR  103 [ OH ] | |
| 3 | D:TYR  42 [ OH ] | | 2.79 | G:LYS 116 [ O  ] | 3x | | 3 | D:ARG  44 [ NH2] | | 3.42 | G:TYR  103 [ OH ] | |
| 4 | D:GLN  44 [ OE1] | | 3.34 | G:TYR  103 [ OH ] | | | 4 | D:THR  49 [ OG1] | | 3.46 | G:ASP 120 [ O  ] | |
| 5 | D:LYS 114 [ O  ] | 3x | 3.30 | G:TRP  107 [ NE1] | 3x | | 5 | D:GLN 114 [ NE2] | 3x | 2.66 | G:GLU  37 [ OE2] | 1x |
| 6 | D:GLU  108 [ O  ] | 3x | 3.40 | G:TYR 114 [ OH ] | 3x | | 6 | D:LEU 116 [ N  ] | 3x | 3.22 | G:TYR  42 [ OH ] | |
| 7 | D:LYS 114 [ O  ] | 3x | 3.04 | G:LYS 116 [ NZ ] | 3x | | 7 | D:GLN 114 [ O  ] | 3x | 3.89 | G:HIS  40 [ NE2] | |
| | | | | | | | 8 | D:GLN 114 [ O  ] | 3x | 3.30 | G:TRP  107 [ NE1] | 3x |
| | | | | | | | | | | | | |
| | 5R1D8\_RTV2M6.B13 | | | | | | | 5R1D15\_RTV2M6.B10 | | | | |
| 1 | D:TYR  42[ OH ] | | 2.92 | G:LYS 116[ O  ] | 3x | | 1 | D:TYR  42[ OH ] | | 2.88 | G:LYS 116[ O  ] | 3x |
| 2 | D:SER  66[ OG ] | | 3.83 | G:THR 113[ OG1] | 3x | | 2 | D:ARG  44[ NH1] | | 3.47 | G:TYR  103[ OH ] | |
| 3 | D:LEU 116[ N  ] | 3x | 3.04 | G:TYR  42 [ OH ] | | | 3 | D:ARG  55[ NH1] | | 2.87 | G:TYR 114[ O  ] | 3x |
| 4 | D:GLN  44[ OE1] | | 3.78 | G:TYR  103[ OH ] | | | 4 | D:THR  66[ OG1] | | 3.46 | G:THR 113[ OG1] | 3x |
| 5 | D:LYS 114[ O  ] | 3x | 3.18 | G:LYS 116[ NZ ] | 3x | | 5 | D:LEU 116[ N  ] | 3x | 2.89 | G:TYR  42[ OH ] | |
| 6 | D:LYS 114[ O  ] | 3x | 3.14 | G:TRP  107[ NE1] | 3x | | 6 | D:GLN 114[ O  ] | 3x | 3.21 | G:TRP  107[ NE1] | 3x |
| | | | | | | | 7 | D:GLN 114[ O  ] | 3x | 3.16 | G:LYS 116[ NZ ] | |
| | | | | | | | | | | | | |
| | 5R1D8\_RTV2M9.B12 | | | | | | | 5R1D15\_RTV2M9.B17 | | | | |
| 1 | D:TYR  42[ OH ] | | 2.97 | G:LYS 116[ O  ] | 3x | | 1 | D:LEU 116[ N  ] | 3x | 3.20 | G:TYR  42[ OH ] | |
| 2 | D:LEU 116[ N  ] | 3x | 3.21 | G:TYR  42[ OH ] | | | 2 | D:ARG  44[ NH1] | | 3.75 | G:TYR  103[ OH ] | |
| 3 | D:LYS 114[ O  ] | 3x | 3.43 | G:TRP  107[ NE1] | 3x | | 3 | D:ARG  55[ NH1] | | 3.16 | G:TYR 114 [ O ] | 3x |
| 4 | D:LYS 114[ O  ] | 3x | 3.85 | G:LYS 116[ NZ ] | 3x | | 4 | D:TYR  42[ OH ] | | 2.94 | G:LYS 116 [ O ] | 3x |
| 1 | D:GLU  49[ OE1] | | 3.93 | G:LYS 123[ NZ ] | | | 5 | D:GLN 114[ O  ] | 3x | 3.20 | G:TRP  107[ NE1] | 3x |
| | | | | | | | 6 | D:GLN 114[ O  ] | 3x | 3.61 | G:LYS 116[ NZ ] | 3x |
| | 1HXM\_AB | | | | | | | | | | | |
| 1 | D:ASP 105[ O  ] | x | 2.90 | G:TRP 100[ NE1] | x | | | | | | | |
| 2 | D:ASP 105[ OD2] | x | 2.64 | G:LYS 109[ NZ ] | x | | | | | | | |
| 3 | D:ASP 105[ O  ] | x | 3.28 | G:LYS 111[ NZ ] | x | | | | | | | |
| 4 | A:LEU 107[ N  ] | x | 2.92 | G:TYR  38[ OH ] | | | | | | | | |
| 5 | D:LYS  40[ NZ ] | | 3.28 | G:GLU  40[ OE1] | | | | | | | | |
| 6 | D:LYS  40[ NZ ] | | 3.40 | G:GLU  40[ OE2] | | | | | | | | |
| 7 | D:TYR  91[ OH ] | | 3.86 | G:GLU  40[ OE2] | | | | | | | | |
| 8 | D:LYS 106[ NZ ] | x | 3.14 | G:GLU  61[ OE1] | | | | | | | | |
| 9 | D:LYS 106[ NZ ] | x | 3.27 | G:GLU  61[ OE2] | | | | | | | | |
| 10 | D:LYS  40[ NZ ] | | 2.89 | G:TYR  96[ OH ] | | | | | | | | |
| 11 | D:TYR  38[ OH ] | | 2.64 | G:LYS 111[ O  ] | x | | | | | | | |
| 1 | D:ASP 105[ OD2] | x | 2.64 | G:LYS 109[ NZ ] | x | | | | | | Salt bridge | |
| 2 | D:LYS  40[ NZ ] | | 3.28 | G:GLU  40[ OE1] | | | | | | | | |
| 3 | D:LYS  40[ NZ ] | | 3.40 | G:GLU  40[ OE2] | | | | | | | | |
| 4 | D:LYS 106[ NZ ] | x | 3.14 | G:GLU  61[ OE1] | | | | | | | | |
| 5 | D:LYS 106[ NZ ] | x | 3.27 | G:GLU  61[ OE2] | | | | | | | | |
B
